# *THEM6*: A Novel Molecular Biomarker Predicts Tumor Microenvironment, Molecular Subtype, and Prognosis in Bladder Cancer

**DOI:** 10.1155/2022/7147279

**Published:** 2022-07-21

**Authors:** Xu Zhao, Jiao Hu, Jiuyi Li, Lan Gu, Jinbo Chen, Belaydi Othmane, Guanghui Gong, Junbin Yuan, Huiyin Deng

**Affiliations:** ^1^Department of Urology, Xiangya Hospital, Central South University, Changsha, 410008 Hunan, China; ^2^Department of Urology, Guizhou Provincial People's Hospital, Guiyang 550001 Guizhou, China; ^3^Department of Anesthesiology, The First People's Hospital of Chenzhou, Chenzhou, 423099 Hunan, China; ^4^Department of Blood Transfusion, The Third Xiangya Hospital, Central South University, Changsha, 410008 Hunan, China; ^5^Department of Pathology, Xiangya Hospital, Central South University, Changsha, 410008 Hunan, China; ^6^Department of Anesthesiology, The Third Xiangya Hospital, Central South University, Changsha, 410008 Hunan, China

## Abstract

**Background:**

Thioesterase superfamily member 6 (*THEM6*) has been implicated in the development and progression of various cancers. However, prior research emphasized on its regulatory role merely, we aim to investigate the effect of *THEM6* gene on the immunological role and its relationship with molecular subtype in bladder cancer (BLCA).

**Methods:**

Through pan-cancer analysis, we explored the *THEM6* expression pattern and immunological role using The Cancer Genome Atlas (TCGA) database. In addition, we performed a correlation of *THEM6* and its immunological functions, including immunomodulators, immune checkpoints, cancer immunity cycles, T cell inflamed score, and tumor-infiltrating immune cells in the BLCA tumor microenvironment (TME) based on TCGA and BLCA microarray cohort from Xiangya Hospital. We also assessed the accuracy of *THEM6* in predicting the molecular subtype and its response to different interventions in BLCA. Finally, we computed and validated a prediction model established by *THEM6*-related different expressed immune-related genes that might help in BLCA prognosis.

**Results:**

*THEM6* led to immunosuppression in BLCA TME. Furthermore, there was a downregulation in the immunological functions. Besides, *THEM6* could effectively distinguish BLCA molecular subtypes, and *THEM6* low expression implied basal subtype that was more effective to several interventions, such as immune checkpoint blockade (ICB) therapies, neoadjuvant chemotherapy, and radiotherapy. While *THEM6* high expression indicated luminal subtype, hyperprogression and better response to targeted therapies, such as blocking *THEM6* and several immune-inhibited oncogenic pathways.

**Conclusions:**

*THEM6* may be with potential immune-modulating properties and may become a potential new immunotherapy target for BLCA. *THEM6* could accurately predict the molecular subtype of BLCA, which was helpful for guiding the treatment. Simultaneously, the prediction model may exhibit an excellent predictive value in patients with BLCA.

## 1. Background

Bladder cancer (BLCA) is the second most common cancer of the urinary tract, with high morbidity and mortality [1]. Despite some progress, the treatment of BLCA, especially advanced BLCA, still has some limitations. Recently, the immune response has been reported to be increasingly associated with the development and management of BLCA [2]. Immunotherapy, such as immune checkpoint blockade (ICB), has been reported to benefit patients with advanced BLCA [3]. With the discovery of immune checkpoints and successful therapeutic outcomes by using immunosuppressants [4], the focus of cancer therapy has shifted from the tumor itself to the host's immune system. The host's anticancer immunity and inflammatory tumor microenvironment (TME) play essential roles in achieving a successful application of ICB therapy [5, 6]. A recent significant breakthrough in tumor immunotherapy is the discovery of adaptive mechanisms of tumor resistance in TME, which may prevent the implementation of tumor immunity [7, 8]. In general, immune and stromal cells are two vital nontumor cells in TME [9–11]. A high number of stromal tumor-infiltrating lymphocytes in tumor immunity in the microenvironment (TIME) suggested an inflammatory subtype, which could predict a disease-specific survival rate of 80% for 5 years, while tumors lacking immune lymphocyte infiltration, called noninflamed subtype, had a survival rate of less than 25% [12]. Based on this background, the conversion of noninflamed TME to inflammatory TME may be crucial for immunotherapy in BLCA, and there is a need for further identification of more biomarkers associated with ICB resistance in BLCA. *THEM6*, also known as C8orf55, encodes for thioesterase superfamily member 6 and is located on chromosome 8q24.3. *THEM6* is a protein-coding gene, which is predicted to have a transmembrane domain protein at the N-terminal. Using tissue microarray (TMA), one prior study reported significant expression of *THEM6* in various cancer tissues in comparison to normal tissues. Besides, its expression was also increased with the progression of cancer, signifying *THEM6* as a potential candidate biomarker for some types of cancer [13].

In our study, through pan-cancer analysis, a significant association of *THEM6* gene with TIME was found, showing substantial negative correlation with BLCA TME, primarily noninflamed TME. While there is still no relevant research on the role of *THEM6* in BLCA, in accordance with the genomic expression profiles, BLCA has been classified into luminal and basal molecular subtype [14]. Molecular subtypes have been reported to have the capability to predict the clinical behaviors and therapeutic effect of BLCA [15–18]. The therapeutic response of the basal subtype to neoadjuvant chemotherapy and ICB is generally better than other subtypes, among which the luminal subtype shows the worst outcome [19].

To evince the function of *THEM6* in TME and its relationship with molecular subtypes in BLCA, our study analyzed the data obtained from authoritative online databases and our hospital. Finally, a prognostic model was designed for predicting the prognosis of BLCA through the algorithm based on machine learning.

## 2. Methods

### 2.1. Data Extraction

TCGA database (https://cancergenome.nih.gov/) was visited online to obtain somatic mutation pan-cancer RNA sequencing (RNA-seq) and survival information data. *THEM6* expression in various cancer cell lines and normal tissues was obtained from Cancer Cell Line Encyclopedia (CCLE) and Genotype-Tissue Expression (GTEx) databases, respectively. Likewise, datasets GSE31684, GSE13507, GSE70691, and GSE32894 (validation cohorts for BLCA) were downloaded from NCBI Gene Expression Omnibus (GEO, https://www.ncbi.nlm.nih.gov/geo/). Meanwhile, our work was continued based on a Xiangya cohort from Xiangya Hospital. Abbreviations of 33 cancer types are shown in Table S1.

### 2.2. Immune-Related Functions of TME in BLCA

We summarized 122 immunomodulators derived from the TISIDB algorithm [20]. Other immunological characteristics (including tumor-immunity activity, immune checkpoints, tumor-infiltrating immune cells, and T cell inflamed score) and corresponding algorithms have also been described, as shown in our prior research [21].

The TIMER database was visited to download the score of immune infiltrating cells. Furthermore, eight independent algorithms (TIMER, Cibersort-ABS, quanTIseq, EPIC, XCELL, MCP-counter, TISIDB, and TIP) were harnessed to conclusively quantify the infiltration level of tumor-infiltrating immune cells (TIICs) leveraged from the bulk of RNA-sequencing data [22–25]. Numerous immune checkpoints (*n* = 20) with therapeutic potential were collected from previous research [26]. Lastly, T cell-inflamed score, which was identified to predict the response of ICB therapy, was calculated using the T cell-inflamed score algorithm [27]. In our study, a linear model was constructed based on a plethora of genes (*n* = 18) along with their coefficients' individual sample T cell-inflamed score.

### 2.3. RNA Sequencing and Analysis

A total of 60 BLCA samples and 12 paired adjacent normal tissues (Xiangya cohort) were obtained from Xiangya Hospital. Clinicopathological information of the cohort was available in Table S2. All of the fresh tissues collected were preserved in liquid nitrogen for total RNA extraction by the Trizol method (Invitrogen, Carlsbad, CA, USA) and quantified through NanoDrop and Agilent 2100 Bioanalyzer (Thermo Fisher Scientific, Massachusetts, USA). Then, we constructed the mRNA library and further purified the total RNA for subsequent segmentation. After that, an amount of about 1 *μ*g of RNA was transcribed into first-strand and second-strand cDNA (Cat #k1622, Thermo Fisher Scientific), and through PCR amplification, single-stranded circular DNA library was constructed. Three of the 60 samples were examined to be unqualified and were excluded from the subsequent analysis. Sequencing was carried out on the BGISEQ-500 platform (BGI, Shenzhen, China), and further analysis was analyzed with TPM values.

### 2.4. Prediction of the Molecular Subtypes of BLCA

Based on previous research, 7 different molecular subtype classification methods are classified as follows: CIT, Lund, MDA, TCGA, Baylor, UNC, and Consensus subtypes [28–31]. Despite the presence of different numbers of subtypes in these classification methods, there was a strong consistency among them via checking corresponding properties [32, 33]. On the basis of the correlations in distinct molecular subtype systems, BLCA could be grouped into two major categories, basal and luminal subtypes [28]. Likewise, an association was established concerning *THEM6* gene with distinct molecular subtypes and characteristic gene signatures related to BLCA. Finally, receiver operating characteristic (ROC) curves were plotted for an accurate prediction of *THEM6* for molecular subtypes.

### 2.5. Enrichment Analysis of Different Gene Therapeutic Signatures

A set of gene signatures was involved for enrichment analysis, which has been confirmed to have a positive correlation with atezolizumab (an anti-PD-L1 agent) as mentioned in a prior study [34]. Similarly, 12 BLCA signatures that are representative of distinct molecular subtypes were included from the International Bladder Cancer Network (IBCN) [28]. Relevant therapeutic signatures were obtained from the DrugBank database.

### 2.6. BLCA Microarray and Histological Ascertainment

For microarray analysis, 60 BLCA samples mentioned above were collected from Xiangya Hospital to prepare TMA samples (1.5 cm in diameter). For immunohistochemistry (IHC) studies, IHC staining was performed using primary antibodies CD8 (ab4055, Abcam), PD-L1 (ab213524, Abcam), and *THEM6* (NBP1-84052, Novus) for incubation at 4°C overnight. After that, the processed tissues were then incubated with corresponding secondary antibodies for 1 h at 37°C. Following washing with phosphate-buffered saline (PBS), samples were treated with diaminobenzidine (DAB) solution and hematoxylin, washed and dehydrated, and then fixed on glass slides.

In order to further elucidate CD8+ T cell immune phenotype along with PD-L1/*THEM6* expression, these tumor phenotypes were further grouped on the basis of CD8+ T cell distribution within different compartments of parenchyma and stroma. Phenotypes such as “inflamed phenotype” comprises CD8+ T cells that are only confined to the parenchymal compartment, while the “excluded phenotype” consists of CD8+ T cells only in the stromal compartment, with an absence of CD8+ T cells concerning the “deserted phenotype” in both parenchyma and stroma compartments. In this study, we defined the percentage of CD8- and *THEM6-*positive cells with a strong membrane staining (brown staining). PD-L1 scoring was performed in accordance with the protocol as previously described [21]. All slides were determined for at least three different tumor areas. The assessment was completed by two pathologists with a random checkup by an investigator after a period of at least a few weeks.

### 2.7. RT-qPCR

Total RNA (1 *μ*g) of three human BLCA cell lines (T24, J82, and 5637) and one normal human urothelial cell line SV-HUC-1 were isolated and reversely transcribed as described above. All cell lines used in this experiment were purchased from the Institute of Cell Biology, Shanghai, China. Primers used for detecting the differential expression of *THEM6* in cancer and normal cell lines are given: (forward primer: 5′-GCAGCACTGGATCTCCTACAACG-3′, reverse primer: 5′-GGTCCTTGGTGACTCACTGAGC-3′); GAPDH (forward primer: 5′-GAAGGTGAAGGTCGGAGTC-3′, reverse primer: 5′-GAAGATGGTGATGGGATTTC-3′). Amplification was used as a reference gene. Then, TB Green (Takara, Dalian, China) were used for amplifying target cDNA fragments on a ViiA™ 7 Real-Time PCR System (ABI, Carlsbad, CA, USA). *THEM6* expression was calculated using the 2^−*ΔΔ*CT^ method and was normalized to GAPDH.

### 2.8. Identification of Different Expressed Immune-Related Genes (DEIRGs)

Data collected from TCGA database were divided into two groups, respectively, according to the median value of *THEM6* expression, as well as stromal and immune scores. Then, the DEIRGs based on *THEM6* expression were obtained by applying “limma” packages to RNA-seq data. The criteria for DEIRGs were |log (fold change)| > 1 and adjusted *P* value < 0.01, Then the VennDiagram R package was for common DEIRGs (co-DEIRGs). The clusterProfiler of R package was used for Gene Ontology (GO) classification analysis, Kyoto Encyclopedia of Genes and Genomes (KEGG) pathway analysis, and visualization of DEIRGs (*P* < 0.05). Similarly, the Search Tool for the Retrieval of Interacting Genes/Proteins (STRING) software (https://string-db.org) was used to construct protein-protein interaction (PPI) network with an interaction score of ≥0.4.

### 2.9. Computing, Training, and Validation of the Prediction Model

We used BLCA data from TCGA database as training set. In order to develop an immune-related signature based on *THEM6* expression, univariate Cox regression analysis by the survival R package was performed on the co-DEIRGs for BLCA survival-related DEIRGs (*P* < 0.05) in the training set. Afterwards, these DEIRGs were used to compute a prognostic model for BLCA through LASSO regression and multivariate Cox regression analysis to select the best candidate DEIRGs. Further visualization of DEIRGs was realized through the package “glmnet” and “forestplot” in R. According DEIRGs RNA-expression profiles, we computed a prognostic model. The formula was obtained as follows: prediction model = ∑coefficient(*i*)∗RNA(*i*), of which *i* represents the RNA-expression profile of DEIRGs.

In the next step, in order to assess the correlation between clinical parameters and DEIRGs, univariate and multivariate Cox regression analyses were employed to screen clinical indicators with prognostic utility. Then, the ROC curves and area under the curve (AUC) values of BLCA survival outcomes were compared and plotted for clinical indicators (including age, gender, grade, stage, subtype, and LVI) alone (model 1) and clinical indicators combined with DEIRGs (model 2) involved in the prognostic model. The AUC values of two models were compared to determine whether the selected DEIRGs was an independent prognostic factor. Finally, random TCGA training cohort and validation cohort were further divided into high- and low-risk groups based on *THEM6*-related DEIRG prognostic model. Following the effectiveness of the median of both cohorts, the prognostic value of prognostic model was validated using the “survival,” “survminer,” and “survivalROC” in R in both groups. In addition, GSE13507, GSE32894, and GSE70691 downloaded from GEO database were used as the prognostic model external validation set.

### 2.10. Statistical Analysis

Statistical analyses were performed by using R (version 4.2.2). A value of *P* < 0.05 was considered to indicate the presence of a statistically significant difference. RNA expression data from database were log_2_ transformed. The response to ICB was examined using Tumor Immune Dysfunction and Exclusion (TIDE) algorithm. ConsensusMIBC and BLCAsubtyping R packages were used for individual molecular subtypes. All the therapeutic signatures were computed using the GSVA R package for enrichment analysis. BLCA immunological and stromal scores were measured by the ESTIMATE R package. On the basis of low and high expression groups, the Student *t*-test was used for comparing continuous variables with normal distributions, while Mann–Whitney *U* test was used for those with nonnormal distributions. Meanwhile, the chi-square test or Fisher's exact test was used to compare categorical variables. Correlations were explored by Pearson or Spearman coefficients. In addition, Kaplan-Meier analysis was used to calculate the survival rates and log-rank test for comparing the survival curves of different groups.

## 3. Results

### 3.1. Expression and Disease Prognosis Correlation of *THEM6* and Pan-Cancer

**Figure 1:**
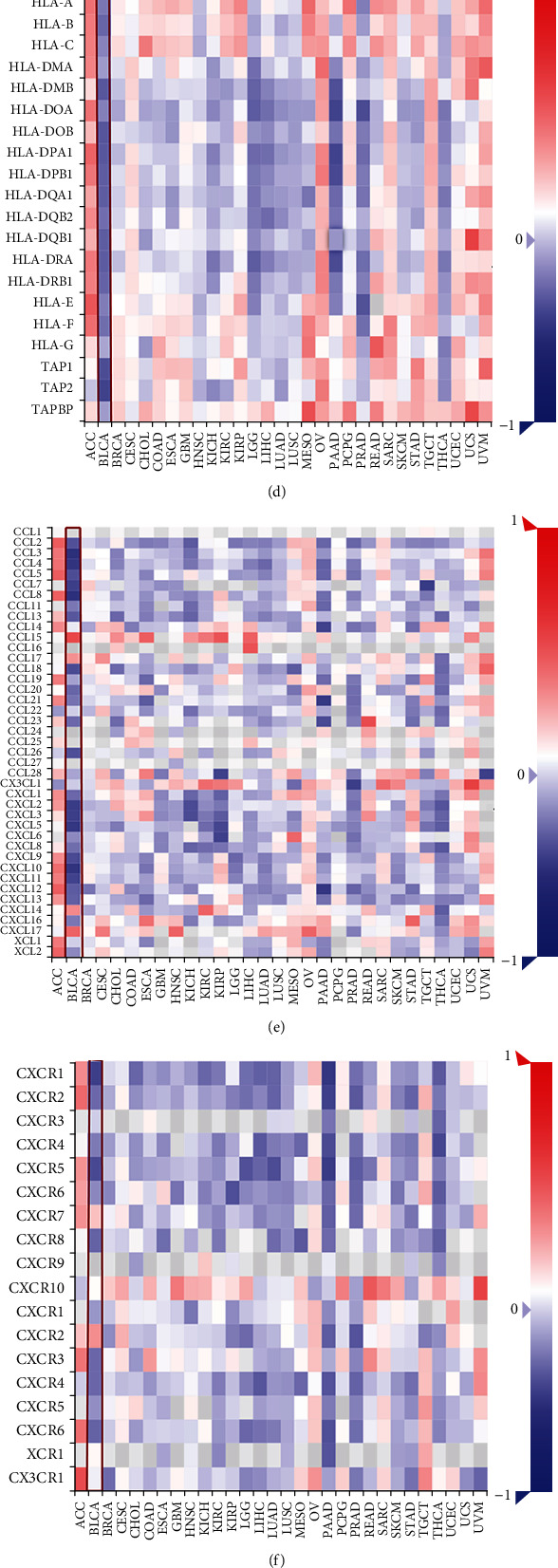
Expression pattern and immunological status of *THEM6* in pan-cancers. (a) The expression pattern of *THEM6* of pan-cancers in TCGA combined with GTEx. The asterisks indicated a significant statistical *P* value calculated with Mann–Whitney *U* test (^∗^*P* < 0.05; ^∗∗^*P* < 0.01; ^∗∗∗^*P* < 0.001). (b) The expression of *THEM6* in cancer cell lines in CCLE. *P* value calculated with Kruskal-Wallis test. (c–f) Correlation between *THEM6* and immunostimulators, MHC molecules, chemokines, and receptors, respectively. (g) Correlation between *THEM6* and 28 tumor-associated immune cells calculated with the ssGSEA algorithm. The color indicates the correlation coefficient, *P* value calculated using Spearman correlation analysis. (h) Correlation between *THEM6* and immune checkpoints. (i–l) Correlation between *THEM6* and four important immune checkpoints, PD-1, PD-L1, CTLA-4, and LAG-3. The dots represent cancer types. The *y*-axis represents the Pearson correlation, while the *x*-axis represents -log10P.

Based on TCGA and GTEx databases, the expression differences of *THEM6* were analyzed among 27 types of cancer and paracancerous tissues (Figure 1(a), Figures S1A and S1B). It was found that there was a higher expression of *THEM6* in most tumor tissues (such as ACC, BLCA, BRCA, CHOL, COAD, ESCA, LIHC, and UCEC) when compared to that in normal paracancerous tissues. In addition, analysis based on the CCLE database discovered that *THEM6* was also highly expressed in many cancer cell lines, such as gastric cancer, prostate cancer, and BLCA (Figure 1(b)). The higher expression of *THEM6* in pan-cancer inspired us to explore its further influence in tumor progression and its impact on disease prognosis. Our subsequent analysis thus focused on the influence of *THEM6* in overall survival (OS) (Figures S1C and S1D). According to the results, the expression of *THEM6* was associated with the progression and prognosis of most types of cancers, including BLCA. However, its specific value remains to be further evaluated using multivariate Cox regression model.

### 3.2. Immunological Correlation of *THEM6* and Pan-Cancer

Pan-cancer analysis showed that the expression of *THEM6* was associated with immunomodulators, such as immunostimulators (Figure 1(c)), MHC molecules (Figure 1(d)), chemokines (Figure 1(e)), and receptors (Figure 1(f)) in most tumors, despite varied correlations among different cancers. Significantly, *THEM6* exhibited the most obvious negative correlation with BLCA. Similarly, *THEM6* showed a correlation with most of 28 lymphocytes in the TME, of which the most significant negative correlation was found with BLCA (Figure 1(g)). Then, the association between *THEM6* and immune checkpoints was evaluated (Figure 1(h)), and four important immune checkpoints were evaluated separately, including PD-1 (Figure 1(i)), PD-L1 (Figure 1(j)), CTLA-4 (Figure 1(k)), and LAG-3 (Figure 1(l)). Consequently, the mutual exclusion of *THEM6* expression and these checkpoints was the most pronounced in BLCA.

In addition, *THEM6* was negatively associated with the immune score (Figure S2) and stromal score (Figure S3) in most cancers. Further correlation analysis was made between *THEM6* gene expression and the score of these immune cells in 33 types of cancers. The results showed that BLCA, BRCA, and LCG were screened to be the three types of tumors with the most significant correlation (Figure S4).

Since *THEM6* was found to exhibit the most obvious immunosuppression in TME in BLCA, our next analysis focused on the influence of *THEM6* expression on BLCA primarily.

### 3.3. The Immunomodulatory Role of *THEM6* in the TIME of BLCA

**Figure 2:**
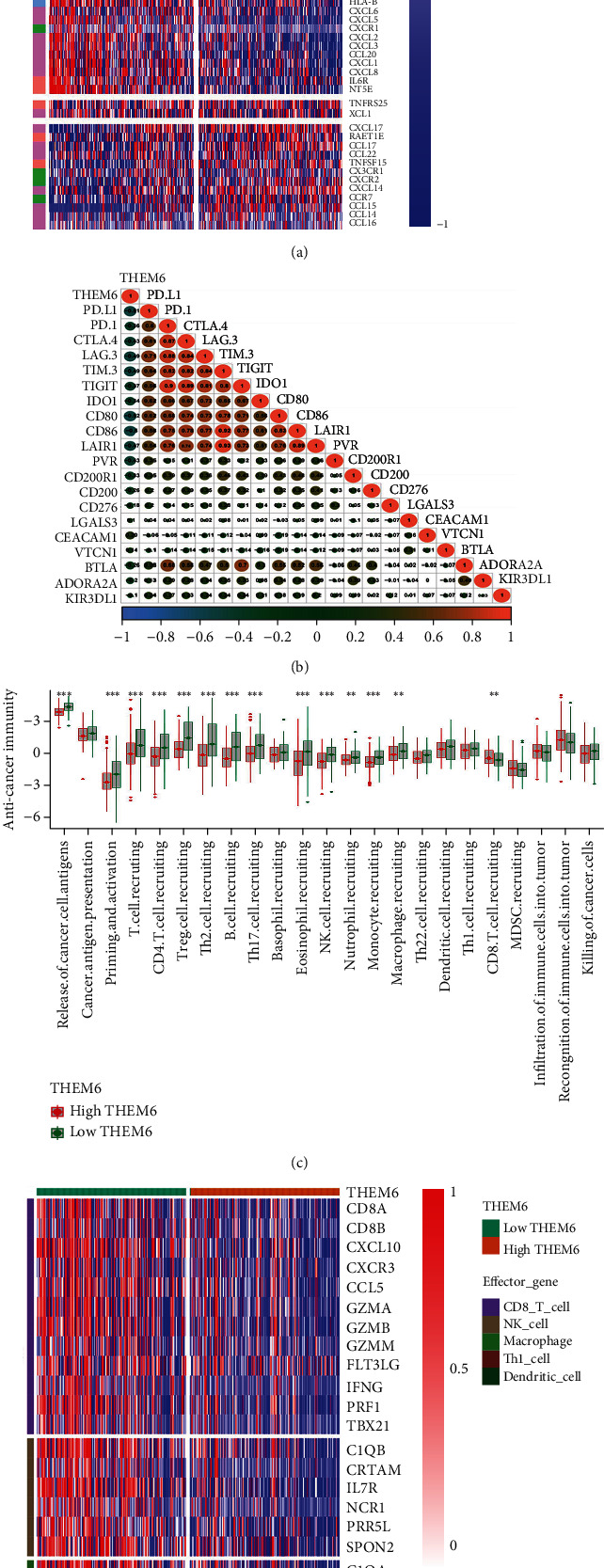
The role of *THEM6* in the BLCA TIME. (a) Differences in the expression of 122 immunomodulators (immunostimulators, MHC molecules, chemokines, and receptors) between the *THEM6*-high and *THEM6*-low groups in BLCA. (b) Correlation between *THEM6* and 20 inhibitory immune checkpoints. (c) Differences in the various steps of the cancer immunity cycle between the *THEM6*-high and *THEM6*-low groups. (d) Differences in the effector genes of the tumor-associated immune cells (CD8+ T cells, NK cells, macrophages, Th1 cells, and dendritic cells) between the *THEM6*-high and *THEM6*-low groups. (e, f) Correlations between *THEM6* and the pan-cancer T cell inflamed score and the individual genes included in the T cell inflamed signature. The color and the values indicate the Spearman correlation coefficient. The asterisks indicated a statistically significant and *P* value calculated using the Mann–Whitney *U* test (^∗^*P* < 0.05; ^∗∗^*P* < 0.01; ^∗∗∗^*P* < 0.001).

The expression of *THEM6* was negatively correlated with most immunostimulators in BLCA, such as *TNFSF9*, *TNFRSF18*, *CD28*, *CD276*, *TNFRSF4*, *TNFRSF17*, and *CD4*. In the *THEM6*-high expression group, there was a downregulation in most of the MHC molecules, which represent antigen presentation and processing ability. Furthermore, chemokines which can induce antigen-presenting cell and TIIC recruitment were downregulated in the *THEM6*-high expression group in BLCA TME, including *CXCL9*, *CXCL10*, *CXCL13*, *CCL2*, *CCL3*, *CCL4*, *CCL11*, *CCL13*, *CCL18*, *CCL19*, *CCL21*, *CCL23*, and *CCL26*. Paired receptors with these chemokines like *CCR1*, *CCR2*, *CCR4*, *CCR5*, *CCR6*, *CCR8*, *CXCR4*, *CXCR5*, and *CXCR6* were also negatively correlated with *THEM6* mRNA expression (Figure 2(a)).

**Figure 3:**
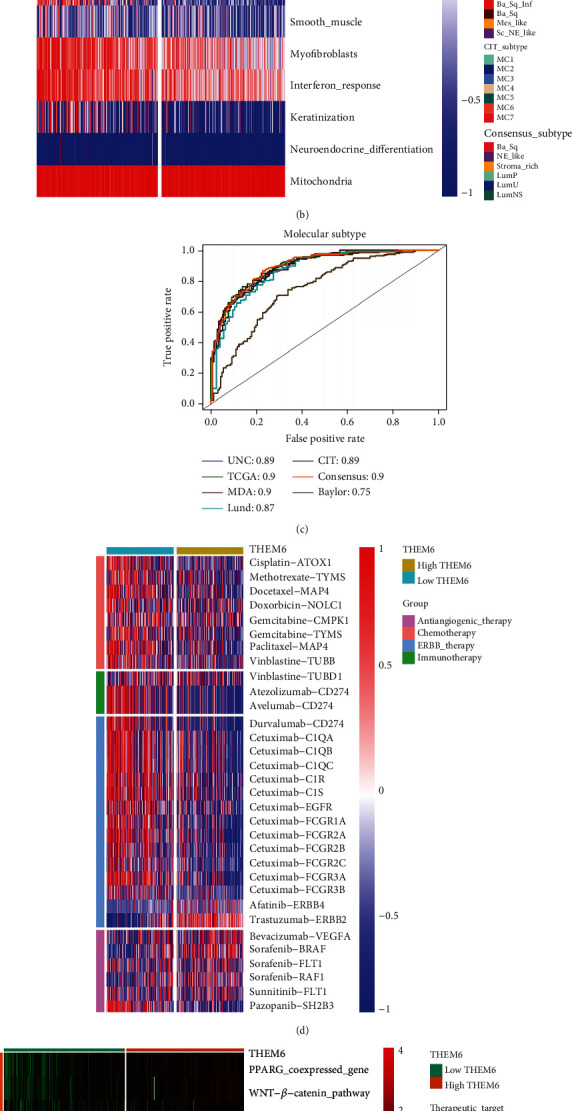
Prediction of the BLCA molecular subtypes and efficacy of immunotherapy by *THEM6*. (a) Correlation between *THEM6* and the infiltration levels of five types of TIICs (CD8+ T cells, NK cells, macrophages, Th1 cells, and dendritic cells), which were calculated using five independent algorithms. (b) Correlations between the *THEM6*-high and *THEM6*-low groups and molecular subtypes using seven kinds of classification methods and bladder cancer signatures. (c) Predictive accuracy of *THEM6* for molecular subtypes using seven different algorithms. The accuracy was equal to the area under the ROC curves. (d) Correlation between *THEM6* and the BLCA-related drug-target genes screened from the DrugBank database. (e) Correlations between *THEM6* and the enrichment scores of several therapeutic signatures such as targeted therapy and radiotherapy. (f) Correlation between *THEM6* and mRNA expression of hyperprogression-associated biomarker in BLCA. The asterisks indicated a significant statistical *P* value calculated with the Mann–Whitney *U* test (^∗^*P* < 0.05; ^∗∗^*P* < 0.01; ^∗∗∗^*P* < 0.001).

Similarly, 20 inhibitory immune checkpoints were also downregulated in the *THEM6*-high expression group (Figure 2(b)). Indeed, the activity of the cancer-immunity cycle can directly and comprehensively present the role of the chemokines' system and immunomodulators [35, 36]. In the *THEM6*-high expression group, there was a downregulation in several major steps in the cancer-immunity cycle, including (1) release of cancer cell antigens, (3) priming and activation, and (4) T cell recruiting (Figure 2(c)). It is conceivable that the downregulation of these key steps would lead to a decrease in the infiltration level of TIICs and a diminution of the killing capacity of tumor cells. Consistently, the effector genes of five tumor-associated TIICs (CD8+ T cells, macrophages, Th1 cells, dendritic cells, and NK cells) were also downregulated in the *THEM6*-high expression group (Figure 2(d)). Meanwhile, *THEM6* was also negatively correlated with T cell inflammatory score (Figure 2(f)) and the individual genes it contained (Figure 2(e)). Higher T cell inflamed score might indicate a better clinical response to immunotherapy, showing a positive correlation. Next, five different algorithms were applied to figure out the infiltration level of the above five important tumor-associated TIICs. Consequently and similarly, there was a negative correlation between *THEM6* expression and TIICs, respectively (Figure 3(a)). Figures S5–S9 show the values of each algorithm in detail.

### 3.4. Prediction of BLCA Molecular Subtypes by *THEM6*

Consistently, the *THEM6*-low expression group showed a higher proportion of the basal subtype, while luminal subtype occurred mainly in the *THEM6*-high expression group. It was consistent with the results of higher expression levels of lymphocyte genes in some studies [14, 37]. Furthermore, the *THEM6*-high expression group had a higher enrichment fraction in the three pathways of luminal differentiation, urothelial differentiation, and TA pathway. In contrast, enrichment scores of the basal and EMT differentiation along with the immune infiltration and interferon response were higher in the *THEM6*-low expression group (Figure 3(b)). In addition, except for Baylor molecular typing system, the AUC values of the other typing systems were all >0.85, which also verified the reliability of our results (Figure 3(c)).

### 3.5. Prediction of the Efficacy of Immunotherapy by *THEM6* in BLCA

The efficacy of antiangiogenic therapy was found to be better in the *THEM6*-high expression group, while there was a superior efficacy of chemotherapy, ERBB therapy, and immunotherapy in the *THEM6*-low expression group (Figure 3(d)). Besides, in terms of the enrichment score in the *THEM6*-high expression group, it was lower concerning *EGFR* ligands and radiotherapy-predicted pathways, but higher in immune-inhibited oncogenic pathways (including *PPARG* coexpressed genes, Wnt/*β*-catenin pathway, *FGFR3* coexpressed genes, *IDH1*, *KDM6b*, and *VEGFA*) (Figure 3(e)). Moreover, in this group, overexpression was found in several tumor oncogenic genes (such as *CCND1*, *MDM2*, *MDM4*, and *DNMT3A*), which positively associated with hyperprogression, which led to ICB-related high progression of the disease. Conversely, tumor suppressor genes (*CDKN2A* and *CDKN2B*), which are negatively correlated with hyperprogression, were found to be underexpressed in the *THEM6*-high expression group (Figure 3(f)).

### 3.6. Xiangya Cohort Validates the Function of *THEM6* in BLCA TME and Subtypes

**Figure 4:**
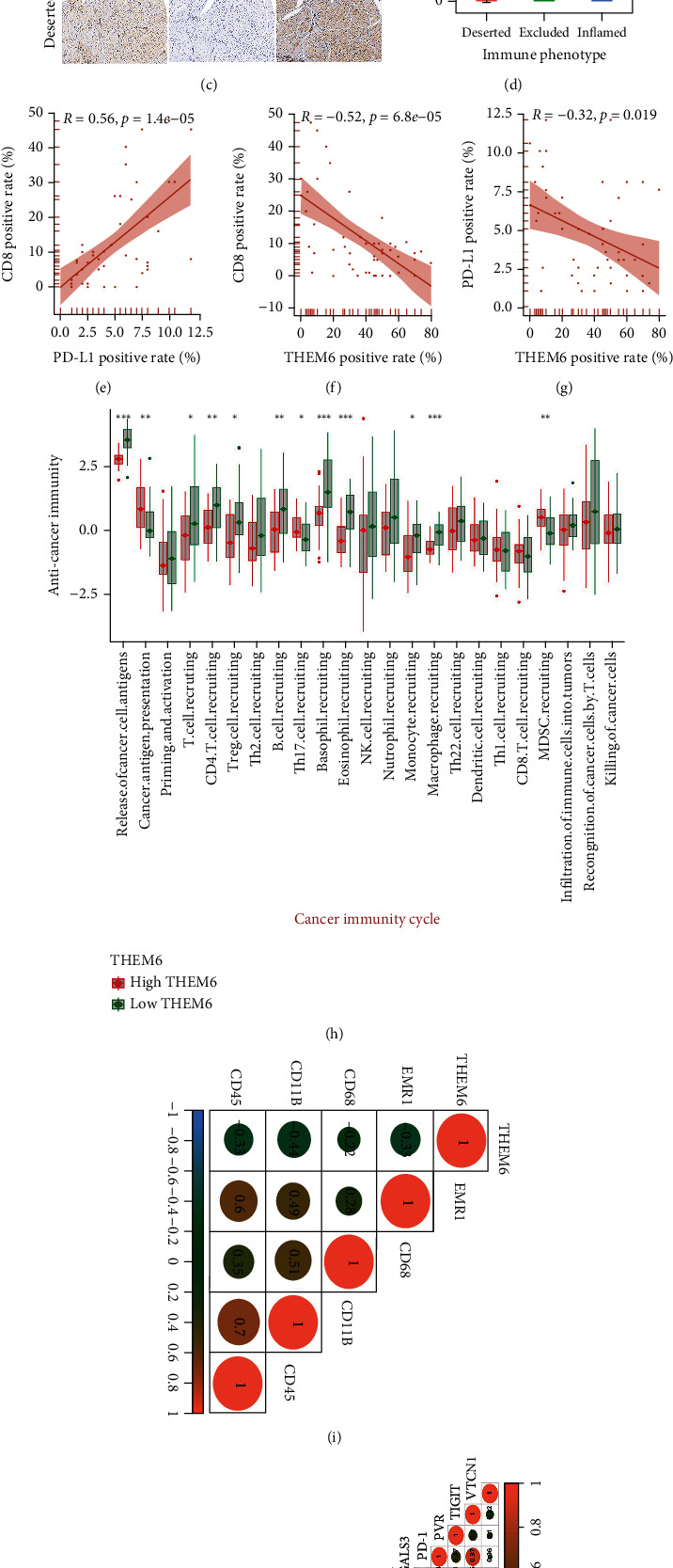
Xiangya cohort validates the relationship between *THEM6* and immune phenotypes. (a) Exome sequencing analysis on *THEM6* mRNA levels in 12 paired bladder cancer and normal tissues. (b) QPCR estimation on three cancer cell lines (T24, J82, and 5637) and one normal cell line (SV-HUC-1). The asterisks indicate a significant statistical *P* value calculated using paired-samples *t*-test. (c) Expression of *THEM6*, PD-L1, and CD8 in the BLCA TMA cohort was detected using immunohistochemistry. Representative images of CD8, PD-L1, and *THEM6* in three immune phenotypes were displayed. The scale bars correspond to 200 *μ*m. (d) CD8-positive rates in the three immune phenotypes in the BLCA TMA cohort detected by IHC. (e) Correlation between PD-L1-positive rates and CD8-positive rates detected using IHC. (f, g) Correlation between *THEM6*-positive rates and CD8/PD-L1-positive rates detected using IHC, respectively. The asterisks indicate a significant statistical *P* value calculated using the Mann–Whitney *U* test (^∗^*P* < 0.05; ^∗∗^*P* < 0.01; ^∗∗∗^*P* < 0.001). (h) Correlations between *THEM6* and the steps of the cancer immunity cycle. (i) Correlations between *THEM6* and four critical marker genes of macrophages. (j) Correlations between *THEM6* and 20 immune checkpoints. The color and the values indicate the Spearman correlation coefficient. IHC: immunohistochemistry.

Firstly, a higher expression of *THEM6* in cancer tissues was verified by exome sequencing analysis of the 12 pairs of matched cancer and adjacent normal tissues (Figure 4(a)). Simultaneously, higher expression of *THEM6* in BLCA cell lines was confirmed compared to normal cell line (Figure 4(b)). IHC showed that CD8+ rate incremented in deserted, excluded, and inflamed types, indicating that this classification method was suitable for the three immune subtypes (Figures 4(c) and 4(d)). Subsequent analysis focused on the relationship among *THEM6*, PD-L1, and CD8. Inflamed was the type with the highest positive rate of CD8, and the expression of PD-L1 was also at its highest, while that of *THEM6* was at its lowest. Besides, the expression of *THEM6* was correlated negatively with CD8 and PD-L1 expression, and PD-L1 showed a positive correlation with CD8 expression (Figures 4(e)–4(g)).

Notably, *THEM6* was correlated negatively with most of the immunomodulators (Figure S10). As for the cancer-immunity cycle, the activity of step 1 (release of cancer cell antigens) and step 4 (T cell recruiting) was downregulated in the *THEM6*-high expression group. However, step 2 (cancer antigen presentation) was upregulated, which might be explained by the increased number of tumor neoantigen caused by the high expression of *THEM6* (Figure 4(h)). Similarly, *THEM6* was also negatively correlated with four important macrophage marker genes (Figure 4(i)) and multiple immune checkpoints (Figure 4(j)).

**Figure 5:**
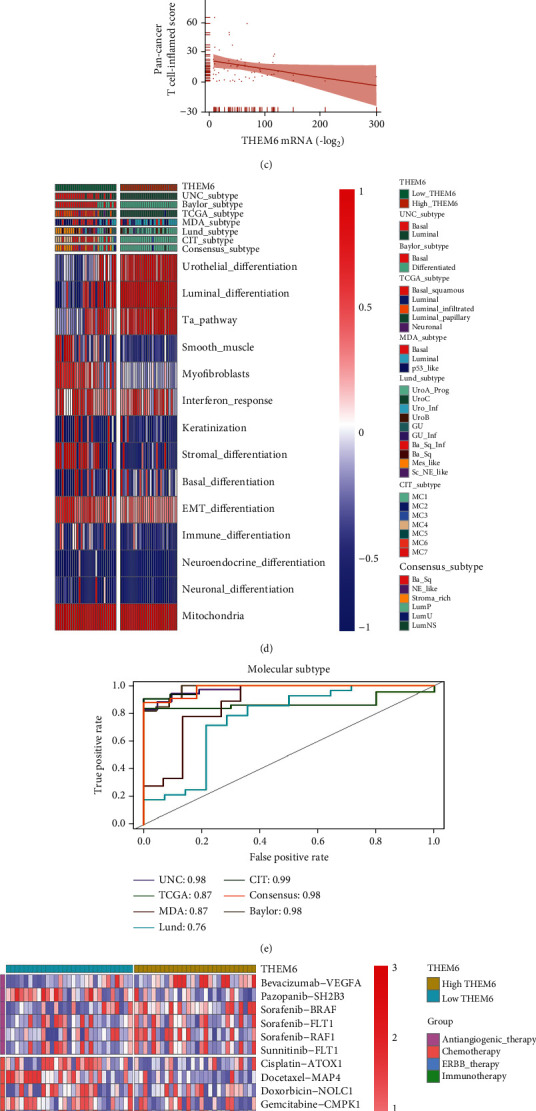
Role of *THEM6* in predicting molecular subtypes and therapeutic sensitivities in Xiangya cohort. (a) Correlation between *THEM6* and the infiltration levels of five types of TIICs (CD8+ T cells, macrophages, Th1 cells, dendritic cells, and NK cells), which were calculated using seven independent algorithms. (b, c) Correlations between *THEM6* and the T cell inflamed score and the individual genes included in the T cell inflamed signature. (d) Correlations between *THEM6*, molecular subtypes, and bladder cancer signatures. (e) ROC curves indicated the predictive accuracy of *THEM6* in predicting molecular subtypes. (f) Correlation between *THEM6* and the BLCA-related drug-target genes screened from the DrugBank database. (g) Correlations between *THEM6* and enrichment scores of therapeutic signatures, including radiotherapy and targeted therapy.

As previously highlighted, there was a consistency with regard to the results of seven different algorithms; *THEM6* was negatively correlated with CD8+ T cells, macrophages, Th1 cells, dendritic cells, and NK cells (Figure 5(a), Figures S11–S17). Meanwhile, *THEM6* showed no obvious correlation with T cell inflammatory score (Figure 5(c)), but was negatively correlated with the individual genes it contained (Figure 5(b)). The difference in this result may be attributed to the quite small sample size. This result was also verified by the GSE31684 database (Figure S18).

In terms of the molecular subtypes of BLCA, the *THEM6*-high expression group was more inclined towards the luminal subtype and its signatures, while the *THEM6*-low expression group towards the basal subtype and its signatures (Figure 5(d)). Significantly, ROC curves displayed in Figure 5(e) verified the accuracy of *THEM6* in predicting BLCA molecular subtypes well. Similarly, BLCA-related drug-target genes, including chemotherapy, ERBB therapy, and immunotherapy genes, revealed negative correlation with *THEM6* mRNA expression (Figure 5(f)). In addition, enrichment scores of the immune-inhibited oncogenic pathways had a positive correlation with *THEM6* mRNA expression (Figure 5(g)). These results were consistent with those identified based on data from TCGA database.

### 3.7. *THEM6*-Related DEIRG Prediction Model of BLCA

A total of 214 genes, 320 genes, and 316 genes were screened out according to the median of *THEM6* expression, stromal score, and immune score, separately. Finally, a total of 80 co-DEIRGs were screened out by the VennDiagram R package (Table S3). Among the 80 genes, no overlap was observed between the *THEM6*-high expression group and the high rating of the immune and stromal score or between the *THEM6*-low expression group and the low rating of the immune and stromal score (Figures S19A–S19E). It, once again, verified the negative correlation between *THEM6* and immunity, as mentioned above. Next, as for the number of protein nodes in the PPI network diagram, about 80 co-DEIRGs are shown in Figure S19F, and several clusters showed intimate association with the immune response. GO and KEGG analyses were further carried out to identify the signal pathways activated by 80 co-DEIRGs. Consequently, these DEIRGs were found to be enriched in immune-related functions (Figures S19G and S19H).

**Figure 6:**
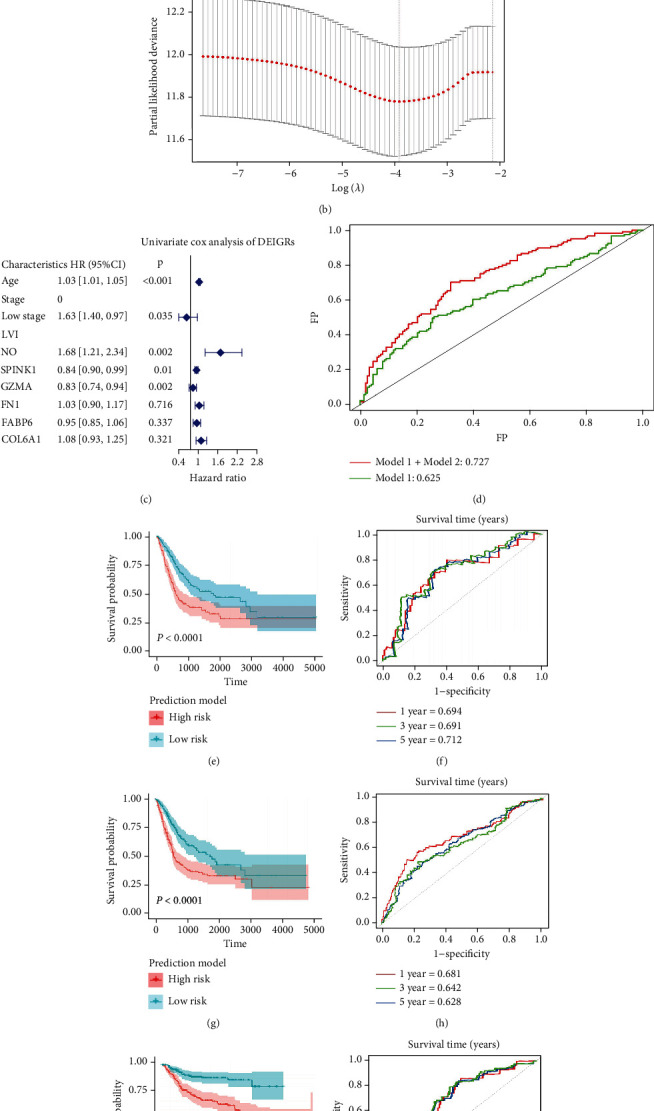
*THEM6*-related DEIRG prediction model of BLCA. (a) LASSO coefficient profiles of 80 prognostic RNAs in TCGA training cohort. The coefficient profile plot was developed against the log (Lambda) sequence. (b) Cross-validation for tuning parameter selection via minimum criteria in the LASSO regression model. Optimal RNAs with the best discriminative capability (6 in number) were selected. (c) Forest plot of the DEIRG RNA-expression profiles and clinic parameters in multivariate Cox analysis. (d) Per-prediction of OS with ROCs from multivariable models. *P* < 0.0001 for difference between models: (green) model 1: age, stage, LVI; (red) model 1 + model 2: age, stage, LVI+*GZMA*, *SPINK1*. (e, f) Development of prediction model and predicting its accuracy for survival in TCGA training set. (g, h) Validation of the prediction model in TCGA validation set. (i, j) Validation of the prediction model in a merged external GEO set (including GSE13507, GSE31684, and GSE70691).

Then, univariate Cox regression analysis was performed on the 80 co-DEIRGs and 23 survival-related DEIRGs were identified to be BLCA survival-related (Table S4). Then, the screened 23 DEIRGs were used for LASSO regression analysis. Finally, two *THEM6*-related DEIRGs (*GZMA* and *SPINK1*) were determined (Figures 6(a) and 6(b)). After that, these two significant variables were verified using forest plot, both of which were confirmed to be independent predictors of clinical prognosis possibly (Figure 6(c)), which had not been reported before. At the same time, univariate Cox regression was used to identify clinical indicators with prognostic value (including age, stage, and LVI) (Table S5). Then, the ROC curves and AUC values of BLCA survival outcomes were compared and plotted for clinical indicators alone (model 1) and clinical indicators combined with the two DEIRGs (model 2). It was found that the AUC value of model 1 and model 2 was 0.625 and 0.725, respectively (Figure 6(d)). In this regard, *GZMA* and *SPINK1* were demonstrated again to be independent prognostic factors. Meanwhile, it suggested that clinical indicators combined with *THEM6*-related DEIRGs had better predictive power and accuracy for BLCA when compared with clinical features alone.

Accordingly, *GZMA* and *SPINK1* were used to construct the *THEM6*-related DEIRG prediction model. The ROC curve and AUC value of the prediction model showed that it had a predictive value for the occurrence of adverse outcomes. In TCGA training set, the OS of the high-risk group was significantly lower than that of the low-risk group (Figure 6(e)). The AUC of the prognostic model was 0.694, 0.691, and 0.712 at 1, 3, and 5 years, respectively (Figure 6(f)). The prediction model formula is [expression level of *GZMA*∗0.180389] + [expression level of *SPINK*1∗(−0.062360)]. Finally, the predictive accuracy of the prediction model for clinical prognosis in BLCA patients was well validated in TCGA validation dataset (Figures 6(g) and 6(h)) and a weighted dataset covering GSE13507, GSE31684, and GSE70691 dataset (Figures 6(i) and 6(j)).

Therefore, the constructed *THEM6*-related DEIRG prognostic model was suitable for predicting clinical outcomes in patients with BLCA, with relatively good accuracy.

## 4. Discussion

Current studies have confirmed the effectiveness of biomarker detection and its function in the prediction of prognosis and survival for BLCA [38, 39]. Our study found that the expression and function of *THEM6* are similar to these biomarkers. In our research, it was also found that *THEM6* was highly expressed in BLCA and correlated with prognosis. Through pan-cancer analysis, clearly, *THEM6* mRNA expression is upregulated in various cancer tissues and most cancer cell lines. Meanwhile, it has been found that high expression of *THEM6* in different cancer types may lead to different clinical outcomes. An earlier study using TMA included 14 tumor tissues and corresponding normal tissues which showed a high expression of *THEM6* in colorectal cancer, gastric cancer, and breast cancer, and it was positively correlated with the progression of these cancers [13]. Both our study and this study have found low expression of *THEM6* in some cancer tissues, but the significance of its low expression has not been studied yet. Recently, Blomme et al. also found that *THEM6* was highly expressed in prostate cancer and associated with poor prognosis [40]. Part of the reason for the interlaboratory variability regarding *THEM6* findings and expression might be that there were heterogeneity and differences in biological behavior between different cancer types. Inspired by the research carried by Francesco et al., the interlaboratory variability might be further addressed via sensitivity analysis, subgroup analysis, and metaregression analysis [41].

In recent decades, immunotherapy has been recognized to be a promising therapeutic choice for many advanced cancers. Our study further explored the role of *THEM6* in tumor immunologic mechanism. In our study, *THEM6* is associated with the downregulation of immunological functions, such as immunomodulators (immunostimulators, MHC, chemokines, and receptors), immune cycle activity, T cell inflammatory score, immune checkpoints, TIICs, and effector genes. *THEM6* has the characteristic of immunosuppression in the BLCA TIME. High expression of *THEM6* may indicate a noninflammatory TME, thereby leading to the promoted progression of BLCA and worse clinical response to immunotherapy. Besides, the expression of *THEM6* was found to be incompatible with the expression of most immune checkpoint inhibitors. This may be partly attributed to the reduced activation of macrophage colony-stimulating factor, while it can modulate the expression of PD-L1. In other words, *THEM6* may act as an independent target and important negative regulator of cytotoxic T cell function to induce lymphocyte death. Previous studies have documented the critical regulatory role of TIME in tumor progression and metastasis [35, 42]. As a target for tumor immunotherapy normalization, molecular candidates shall meet two conditions of TME-specific overexpression and immunosuppressive function [36]. Significantly in our study, *THEM6* was verified to meet these two conditions in BLCA, proving that *THEM6* may be a potential target for BLCA immunotherapy. Moreover, the expression of *THEM6* was mutually exclusive to PD-L1; consequently, targeting *THEM6* may provide an available choice for managing patients with poor immunotherapeutic response related to PD-L1.

In view of the correlation analysis between *THEM6* expression and BLCA molecular subtypes, the *THEM6*-high expression group was more inclined towards the luminal subtype, while the *THEM6*-low expression group towards the basal subtype. Generally, the luminal subtype may indicate a worse effect of immunotherapy. Indirectly, it may imply that high expression of *THEM6* may lead to a reduced effect of ICB, and such patients may not be suitable for ICB treatment. Consistent with our results, it has been reported that BLCA patients might experience a better prognostic outcome when there was high expression of immune checkpoints, high levels of CD8+ inflammatory cell infiltration, and in those who were confirmed with basal subtype of this cancer [43]. Pembrolizumab, a monoclonal antibody, has been used to treat cancer, which exhibits the most significant effect on treating basal subtype in terms of pathology and immune cell infiltration when compared with other molecular subtypes of BLCA [17]. We could infer that pembrolizumab is more suitable for patients with *THEM6 low* expression.

Carcinogenic pathways, which suppress the immune system and consist of *FGFR3*, *β*-catenin, and *PPAR*-*γ* pathways, are reported to form a noninflammatory TME by impairing the infiltration of immune cells and lessening the expression of immunomodulators [44, 45]. Reversing these carcinogenic pathways can reactivate tumor immunity and trigger an anticancer immune response in TME [46, 47]. In our study, *THEM6* has a similar effect to these pathways to a certain extent. Subsequent analysis on treatment pathways also proved that the efficacy of antiangiogenic therapy was better in the *THEM6*-high expression group, *THEM6*-low expression group, corresponding to the basal subtype, gained benefit more significantly from chemotherapy, radiotherapy, *ERBB* therapy, and immunotherapy treatment. Besides, in terms of the enrichment score in the *THEM6*-high expression group, it was higher in classical immune-inhibited oncogenic pathways and several tumor oncogenic genes, which led to ICB-related high progression of the disease. Conversely, tumor suppressor genes were found to be underexpressed in the *THEM6*-high expression group. The immune-inhibited oncogenic pathways were associated with the noninflammatory TME, that was, a blockage of the formation of immune-inhibited carcinogenic pathways can improve the noninflammatory TME resulted by *THEM6*, so as to reactivate the immune system of the tumor and play a therapeutic role. As we predicted, drugs targeting the *FGFR* pathway and the *PPAR*-*γ* pathway are currently on the market and have demonstrated efficacy in BLCA patients [48, 49].

So far, there is still an unclear understanding of the clinical importance of IRGs in BLCA. In our study, *THEM6*-DEIGRs was significantly associated with OS of BLCA in univariate and multivariate Cox analyses. The prediction of clinical survival outcomes was significantly improved when *THEM6*-DEIGRs was added to the prognostic model which included clinical information only. It suggests that the proposed machine learning-based tool may be able to improve the current practice patterns. In our study, two *THEM6*-DEIRGs were found to be closely related to clinical survival outcomes of BLCA, both of which were used to construct a prognostic model to accurately predict different survival outcomes of the patients. In addition, the high-risk group in the prognostic model had a significantly lower OS rate, exhibiting a good validation of the predictive accuracy of the *THEM6*-DEIRG prognostic model. Findings in our study may provide evidence to support our hypothesis that the prognostic model constructed in our research has a high accuracy in predicting clinical survival and response to cancer immunotherapy in patients with BLCA and can be used to differentiate high-risk populations, which may contribute to early intervention, reducing risk factors and improving patient outcomes.

Though *THEM6* has the potential to become a new option for diagnosis and treatment target for BLCA and along with the development of molecular target drugs, the current research progress has entered the preclinical or clinical research stage [50]. Because of the enormous difficulties in assessing toxicity, as well as successful drug delivery systems, the clinical application of *THEM6* inhibitor remains to be further confirmed.

## 5. Conclusions

This study showed that *THEM6* can form a noninflammatory TME, suggesting that the application of *THEM6* inhibitor may recover TME immune response and normalize cancer immunotherapy in bladder cancer. *THEM6* also has good performance in predicting the molecular subtype and ICB therapeutic effect of bladder cancer.

## Data Availability

The datasets used and/or analyzed during the current study are available from the corresponding author on reasonable request.

## Supplementary Materials

Supplementary Materials Figure S1: the expression pattern and prognostic analysis for overall survival of *THEM6* in pan-cancers. (A, B) The expression pattern of *THEM6* of pan-cancers in TCGA and GTEx. The asterisks indicated a significant statistical *P* value calculated with the Mann–Whitney *U* test (^∗^*P* < 0.05; ^∗∗^*P* < 0.01; ^∗∗∗^*P* < 0.001). (C) The prognostic analyses of *THEM6* in pan-cancers using a univariate Cox regression model. Hazard ratio > 1 indicated a risk factor and hazard ratio < 1 represented a protective factor. (D) The prognostic analyses of *THEM6* in pan-cancers using the Kaplan-Meier method and log-rank test. Only cancers in which *THEM6* was a significant prognostic biomarker were shown. Figure S2: the correlation of *THEM6* mRNA expression and immune score in pan-cancers. The *P* value was calculated by estimating the R page. Figure S3: the correlation of *THEM6* mRNA expression and stromal score in pan-cancers. The *P* value was calculated by estimating the R page. Figure S4: immunological correlation of *THEM6* in pan-cancers. Three cancers with the most significant differences in inflammatory cell infiltration in the pan-cancer were identified (including BLCA, BRCA, and LGG). Using the TIMER algorithm, *P* value calculated with the Mann–Whitney *U* test. Figures S5–S9: correlations between *THEM6* and the tumor-associated immune cells calculated with the QuanTIseq algorithm, CIBERSORT-ABS algorithm, TISIDB algorithm, TIMER algorithm, and TIP algorithm, respectively. The *P* value was calculated with the Spearman correlation analysis. Figure S10: correlation between *THEM6* and 122 immunomodulators in Xiangya cohort. The color and the values indicate the Spearman correlation coefficient. Figures S11–S17: correlations between *THEM6* and the tumor-associated immune cells calculated with the TIME algorithm, CIBERSORT-ABS algorithm, QuanTIseq algorithm, xCell algorithm, MCP-counter algorithm, TIP algorithm, and EPIC algorithm, respectively, in the Xiangya cohort. The *P* value was calculated with the Spearman correlation analysis. Figure S18: correlations between *THEM6* and the immunological status in GSE31684. (A) Correlation between *THEM6* and 122 immunomodulators. (B) Correlation between *THEM6* and 20 inhibitory immune checkpoints. The color and the values indicate the Spearman correlation coefficient. (C, D) Correlations between *THEM6* and the pan-cancer T cell inflamed score and the individual genes included in the T cell inflamed signature. (E) Differences in the effector genes of the tumor-associated immune cells (CD8+ T cells, NK cells, macrophages, Th1 cells, and dendritic cells) between the *THEM6*-high and *THEM6*-low groups. *P* value calculated with the Mann–Whitney *U* test. Figure S19: *THEM6*-related different expression immune-related gene (DEIRG) prediction model of BLCA. (A) 80 common DEIRGs shown in the Venn diagram. (B, C) There is no intersection between *THEM6* mRNA expression high group and RNAs upexpressed among immune/stromal score groups. Similarly, there is no intersection between *THEM6* mRNA expression low group and RNAs downexpressed among immune/stromal score groups. (D) There are 26 common RNAs between *THEM6* mRNA expression low group and RNAs upexpressed among immune/stromal score groups. (E) There are 54 common RNAs between *THEM6* mRNA expression high group and RNAs downexpressed among immune/stromal score groups. (F) The PPI network of 80 co-DEIRGs, the number of nodes: 77, number of edges: 119, average node degree: 3.09, local clustering coefficient: 0.379, expected number of edges: 31, and PPI enrichment *P* value: <1.0*e* − 16. (G, H) GO and KEGG analysis of 80 common differentially expressed RNAs. Table S1: abbreviations of cancer types. Table S2: clinicopathological information of Xiangya cohort. Table S3: 80 common differentially expressed RNAs. Table S4: univariate Cox analysis of 80 common differentially expressed RNAs. Table S5: clinical univariable and multivariable Cox regression analysis in TCGA BLCA cohort.
