# Supplementary figures and images for: THEM6: A Novel Molecular Biomarker Predicts Tumor Microenvironment, Molecular Subtype, and Prognosis in Bladder Cancer
Source: Dis Markers. 2022 Jul 21;2022:7147279. doi: 10.1155/2022/7147279 (PMC9334031; doi:10.1155/2022/7147279)

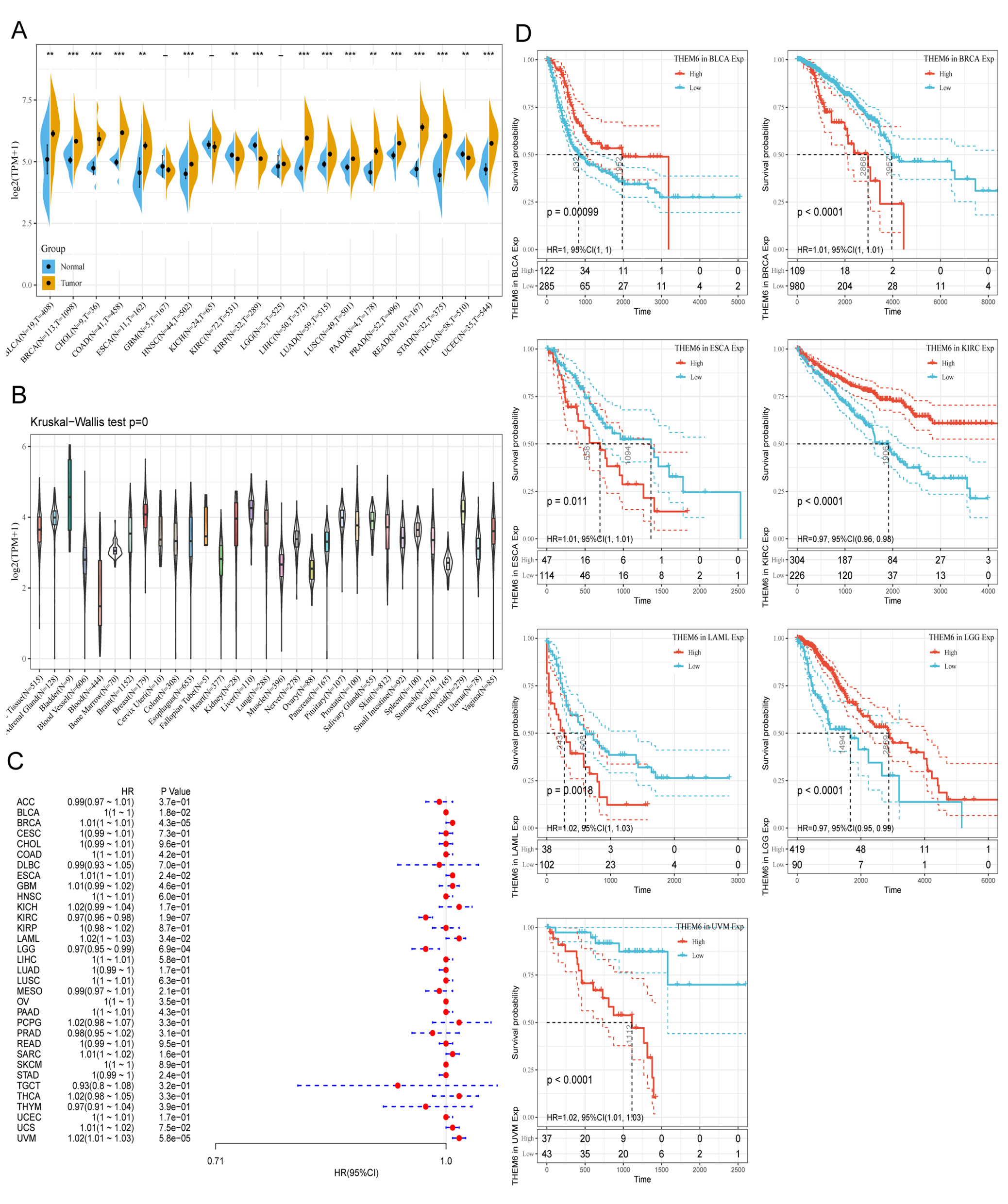

Supplement: Supplementary Materials — Figure S1: the expression pattern and prognostic analysis for overall survival of THEM6 in pan-cancers. (A, B) The expression pattern of THEM6 of pan-cancers in TCGA and GTEx. The asterisks indicated a significant statistical P value calculated with the Mann–Whitney U test (∗P < 0.05; ∗∗P < 0.01; ∗∗∗P < 0.001). (C) The prognostic analyses of THEM6 in pan-cancers using a univariate Cox regression model. Hazard ratio > 1 indicated a risk factor and hazard ratio < 1 represented a protective factor. (D) The prognostic analyses of THEM6 in pan-cancers using the Kaplan-Meier method and log-rank test. Only cancers in which THEM6 was a significant prognostic biomarker were shown. Figure S2: the correlation of THEM6 mRNA expression and immune score in pan-cancers. The P value was calculated by estimating the R page. Figure S3: the correlation of THEM6 mRNA expression and stromal score in pan-cancers. The P value was calculated by estimating the R page. Figure S4: immunological correlation of THEM6 in pan-cancers. Three cancers with the most significant differences in inflammatory cell infiltration in the pan-cancer were identified (including BLCA, BRCA, and LGG). Using the TIMER algorithm, P value calculated with the Mann–Whitney U test. Figures S5–S9: correlations between THEM6 and the tumor-associated immune cells calculated with the QuanTIseq algorithm, CIBERSORT-ABS algorithm, TISIDB algorithm, TIMER algorithm, and TIP algorithm, respectively. The P value was calculated with the Spearman correlation analysis. Figure S10: correlation between THEM6 and 122 immunomodulators in Xiangya cohort. The color and the values indicate the Spearman correlation coefficient. Figures S11–S17: correlations between THEM6 and the tumor-associated immune cells calculated with the TIME algorithm, CIBERSORT-ABS algorithm, QuanTIseq algorithm, xCell algorithm, MCP-counter algorithm, TIP algorithm, and EPIC algorithm, respectively, in the Xiangya cohort. The P value was calculated with the Spea [file 7147279.f1.zip › Figure S1.png]

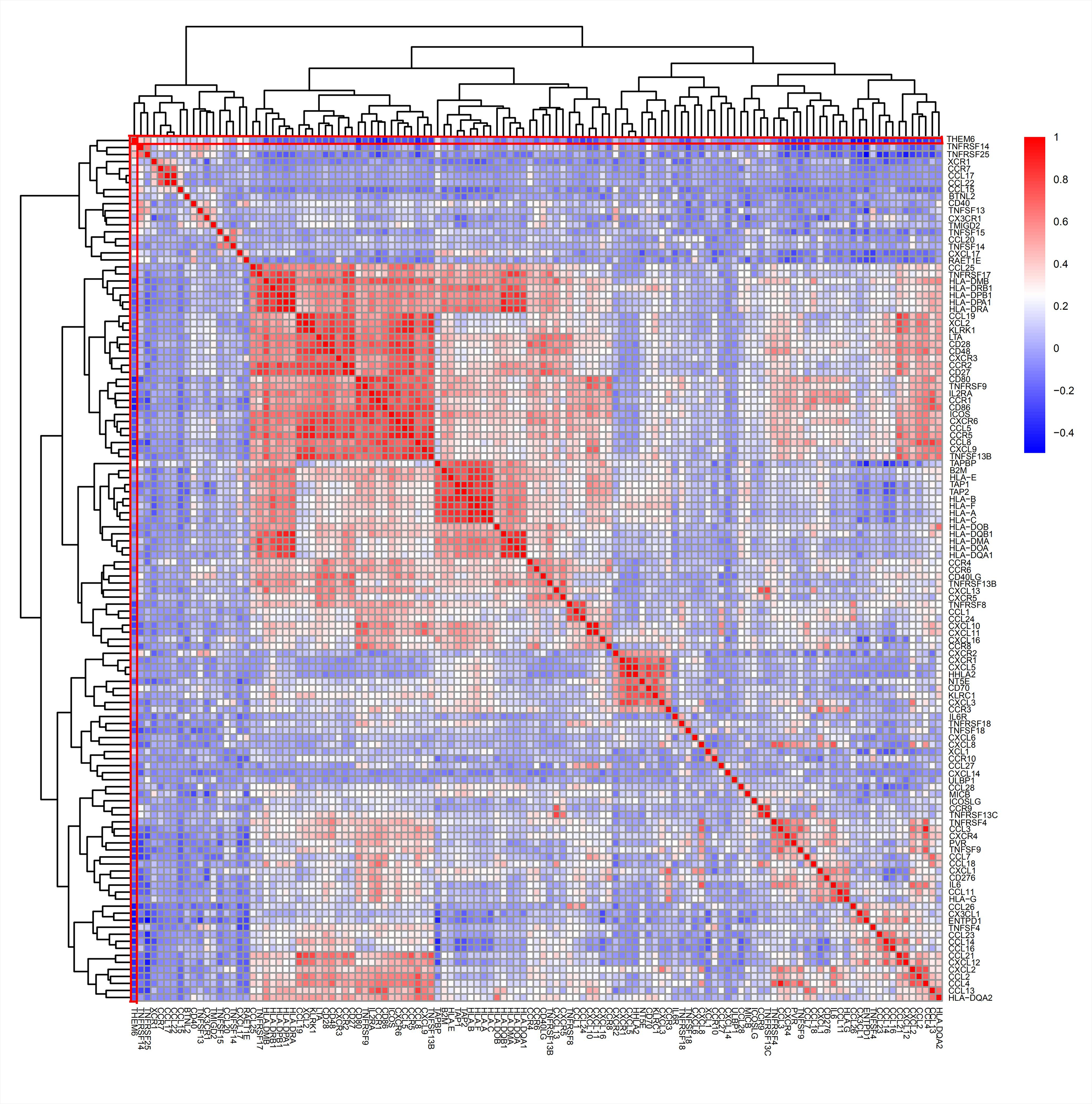

Supplement: Supplementary Materials — Figure S1: the expression pattern and prognostic analysis for overall survival of THEM6 in pan-cancers. (A, B) The expression pattern of THEM6 of pan-cancers in TCGA and GTEx. The asterisks indicated a significant statistical P value calculated with the Mann–Whitney U test (∗P < 0.05; ∗∗P < 0.01; ∗∗∗P < 0.001). (C) The prognostic analyses of THEM6 in pan-cancers using a univariate Cox regression model. Hazard ratio > 1 indicated a risk factor and hazard ratio < 1 represented a protective factor. (D) The prognostic analyses of THEM6 in pan-cancers using the Kaplan-Meier method and log-rank test. Only cancers in which THEM6 was a significant prognostic biomarker were shown. Figure S2: the correlation of THEM6 mRNA expression and immune score in pan-cancers. The P value was calculated by estimating the R page. Figure S3: the correlation of THEM6 mRNA expression and stromal score in pan-cancers. The P value was calculated by estimating the R page. Figure S4: immunological correlation of THEM6 in pan-cancers. Three cancers with the most significant differences in inflammatory cell infiltration in the pan-cancer were identified (including BLCA, BRCA, and LGG). Using the TIMER algorithm, P value calculated with the Mann–Whitney U test. Figures S5–S9: correlations between THEM6 and the tumor-associated immune cells calculated with the QuanTIseq algorithm, CIBERSORT-ABS algorithm, TISIDB algorithm, TIMER algorithm, and TIP algorithm, respectively. The P value was calculated with the Spearman correlation analysis. Figure S10: correlation between THEM6 and 122 immunomodulators in Xiangya cohort. The color and the values indicate the Spearman correlation coefficient. Figures S11–S17: correlations between THEM6 and the tumor-associated immune cells calculated with the TIME algorithm, CIBERSORT-ABS algorithm, QuanTIseq algorithm, xCell algorithm, MCP-counter algorithm, TIP algorithm, and EPIC algorithm, respectively, in the Xiangya cohort. The P value was calculated with the Spea [file 7147279.f1.zip › Figure S10.png]

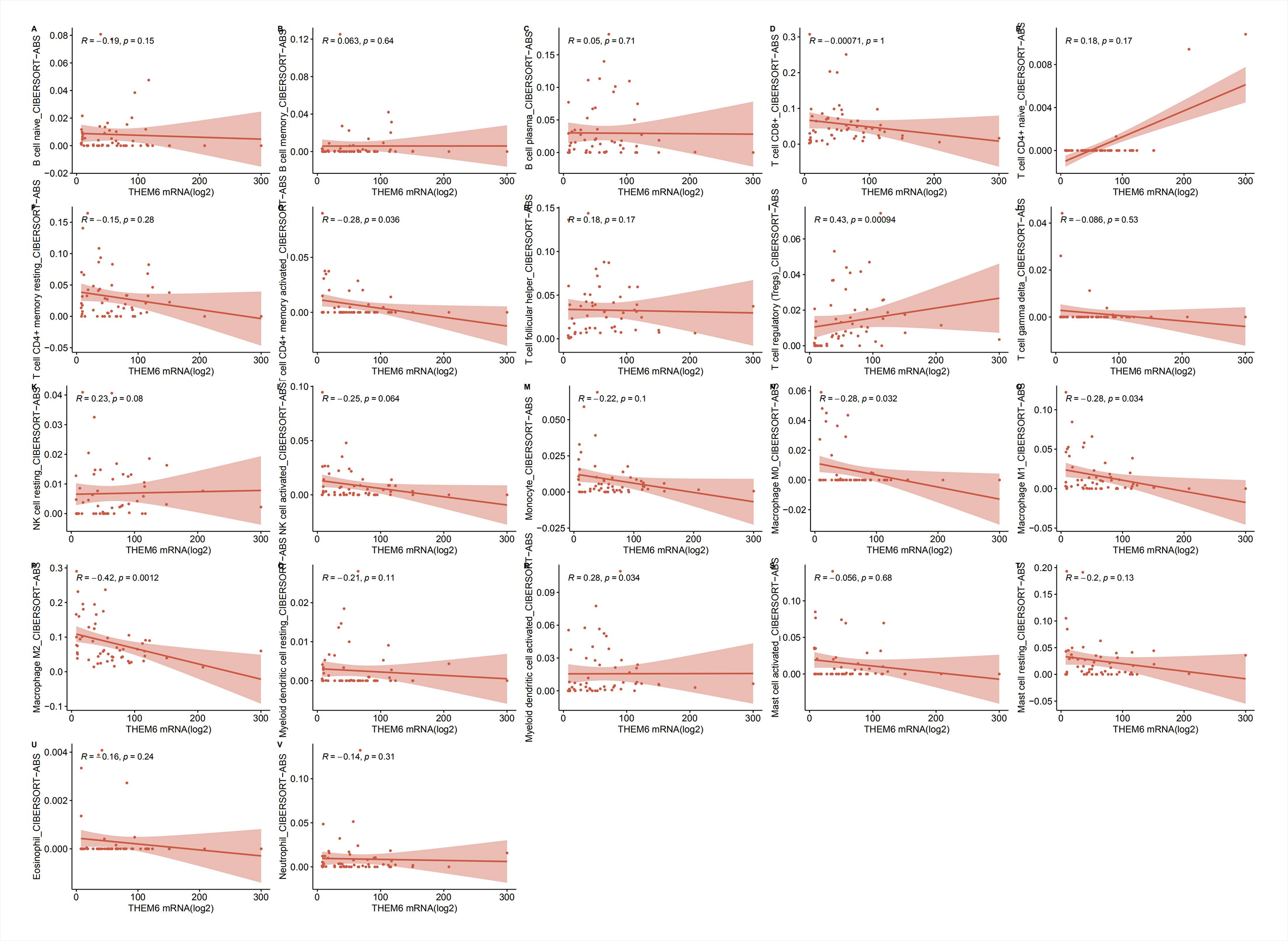

Supplement: Supplementary Materials — Figure S1: the expression pattern and prognostic analysis for overall survival of THEM6 in pan-cancers. (A, B) The expression pattern of THEM6 of pan-cancers in TCGA and GTEx. The asterisks indicated a significant statistical P value calculated with the Mann–Whitney U test (∗P < 0.05; ∗∗P < 0.01; ∗∗∗P < 0.001). (C) The prognostic analyses of THEM6 in pan-cancers using a univariate Cox regression model. Hazard ratio > 1 indicated a risk factor and hazard ratio < 1 represented a protective factor. (D) The prognostic analyses of THEM6 in pan-cancers using the Kaplan-Meier method and log-rank test. Only cancers in which THEM6 was a significant prognostic biomarker were shown. Figure S2: the correlation of THEM6 mRNA expression and immune score in pan-cancers. The P value was calculated by estimating the R page. Figure S3: the correlation of THEM6 mRNA expression and stromal score in pan-cancers. The P value was calculated by estimating the R page. Figure S4: immunological correlation of THEM6 in pan-cancers. Three cancers with the most significant differences in inflammatory cell infiltration in the pan-cancer were identified (including BLCA, BRCA, and LGG). Using the TIMER algorithm, P value calculated with the Mann–Whitney U test. Figures S5–S9: correlations between THEM6 and the tumor-associated immune cells calculated with the QuanTIseq algorithm, CIBERSORT-ABS algorithm, TISIDB algorithm, TIMER algorithm, and TIP algorithm, respectively. The P value was calculated with the Spearman correlation analysis. Figure S10: correlation between THEM6 and 122 immunomodulators in Xiangya cohort. The color and the values indicate the Spearman correlation coefficient. Figures S11–S17: correlations between THEM6 and the tumor-associated immune cells calculated with the TIME algorithm, CIBERSORT-ABS algorithm, QuanTIseq algorithm, xCell algorithm, MCP-counter algorithm, TIP algorithm, and EPIC algorithm, respectively, in the Xiangya cohort. The P value was calculated with the Spea [file 7147279.f1.zip › Figure S11.png]

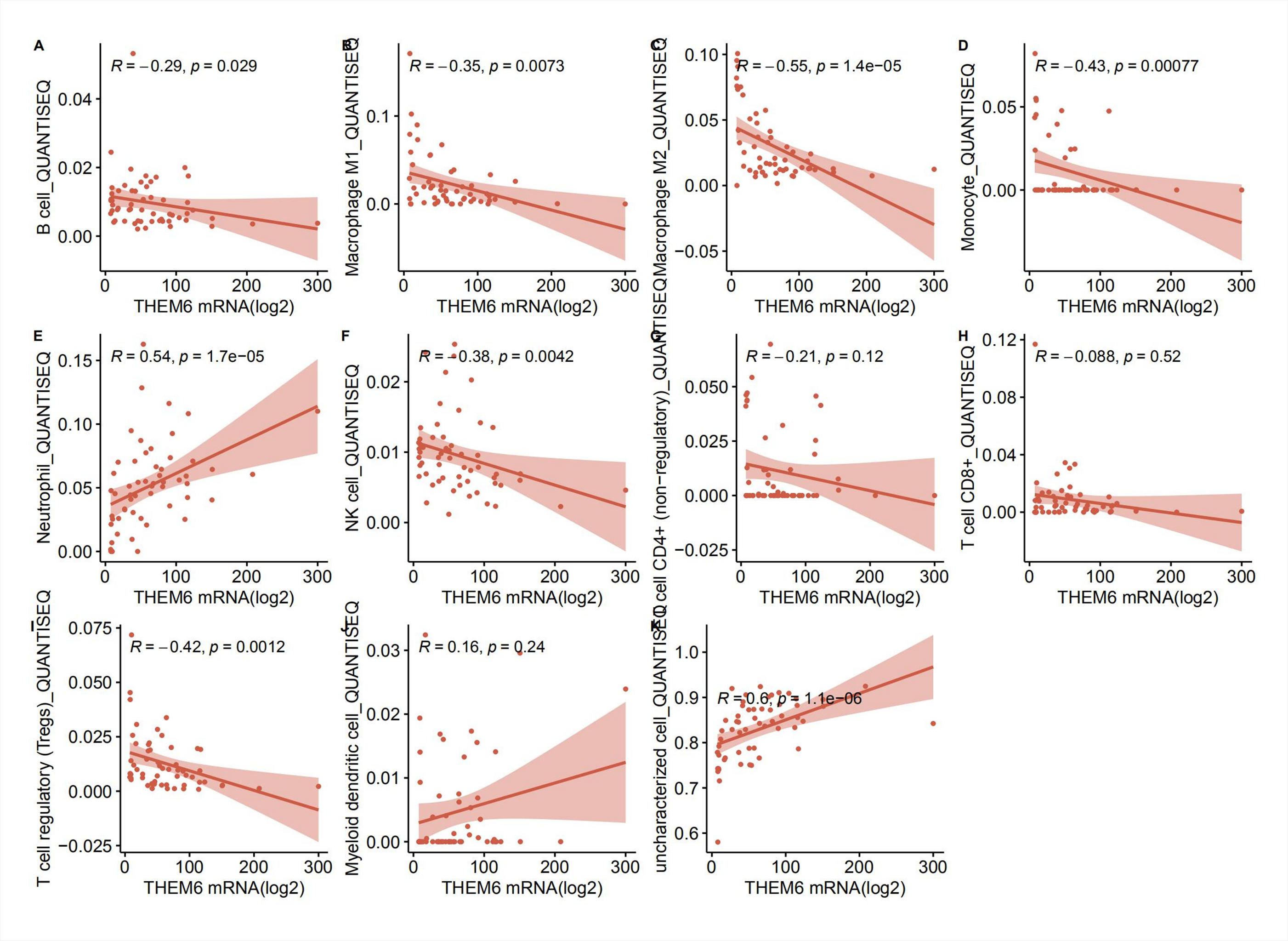

Supplement: Supplementary Materials — Figure S1: the expression pattern and prognostic analysis for overall survival of THEM6 in pan-cancers. (A, B) The expression pattern of THEM6 of pan-cancers in TCGA and GTEx. The asterisks indicated a significant statistical P value calculated with the Mann–Whitney U test (∗P < 0.05; ∗∗P < 0.01; ∗∗∗P < 0.001). (C) The prognostic analyses of THEM6 in pan-cancers using a univariate Cox regression model. Hazard ratio > 1 indicated a risk factor and hazard ratio < 1 represented a protective factor. (D) The prognostic analyses of THEM6 in pan-cancers using the Kaplan-Meier method and log-rank test. Only cancers in which THEM6 was a significant prognostic biomarker were shown. Figure S2: the correlation of THEM6 mRNA expression and immune score in pan-cancers. The P value was calculated by estimating the R page. Figure S3: the correlation of THEM6 mRNA expression and stromal score in pan-cancers. The P value was calculated by estimating the R page. Figure S4: immunological correlation of THEM6 in pan-cancers. Three cancers with the most significant differences in inflammatory cell infiltration in the pan-cancer were identified (including BLCA, BRCA, and LGG). Using the TIMER algorithm, P value calculated with the Mann–Whitney U test. Figures S5–S9: correlations between THEM6 and the tumor-associated immune cells calculated with the QuanTIseq algorithm, CIBERSORT-ABS algorithm, TISIDB algorithm, TIMER algorithm, and TIP algorithm, respectively. The P value was calculated with the Spearman correlation analysis. Figure S10: correlation between THEM6 and 122 immunomodulators in Xiangya cohort. The color and the values indicate the Spearman correlation coefficient. Figures S11–S17: correlations between THEM6 and the tumor-associated immune cells calculated with the TIME algorithm, CIBERSORT-ABS algorithm, QuanTIseq algorithm, xCell algorithm, MCP-counter algorithm, TIP algorithm, and EPIC algorithm, respectively, in the Xiangya cohort. The P value was calculated with the Spea [file 7147279.f1.zip › Figure S12.png]

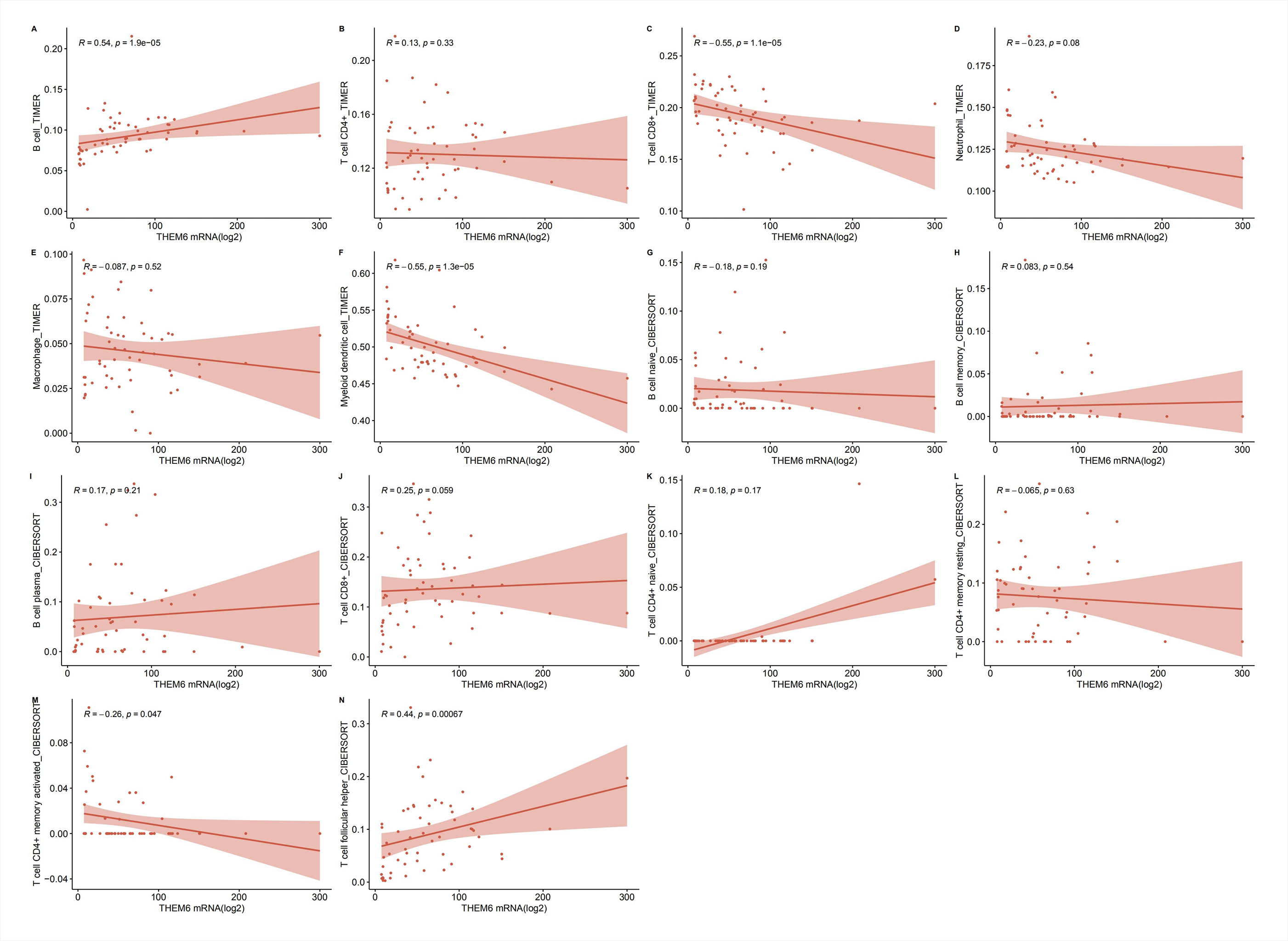

Supplement: Supplementary Materials — Figure S1: the expression pattern and prognostic analysis for overall survival of THEM6 in pan-cancers. (A, B) The expression pattern of THEM6 of pan-cancers in TCGA and GTEx. The asterisks indicated a significant statistical P value calculated with the Mann–Whitney U test (∗P < 0.05; ∗∗P < 0.01; ∗∗∗P < 0.001). (C) The prognostic analyses of THEM6 in pan-cancers using a univariate Cox regression model. Hazard ratio > 1 indicated a risk factor and hazard ratio < 1 represented a protective factor. (D) The prognostic analyses of THEM6 in pan-cancers using the Kaplan-Meier method and log-rank test. Only cancers in which THEM6 was a significant prognostic biomarker were shown. Figure S2: the correlation of THEM6 mRNA expression and immune score in pan-cancers. The P value was calculated by estimating the R page. Figure S3: the correlation of THEM6 mRNA expression and stromal score in pan-cancers. The P value was calculated by estimating the R page. Figure S4: immunological correlation of THEM6 in pan-cancers. Three cancers with the most significant differences in inflammatory cell infiltration in the pan-cancer were identified (including BLCA, BRCA, and LGG). Using the TIMER algorithm, P value calculated with the Mann–Whitney U test. Figures S5–S9: correlations between THEM6 and the tumor-associated immune cells calculated with the QuanTIseq algorithm, CIBERSORT-ABS algorithm, TISIDB algorithm, TIMER algorithm, and TIP algorithm, respectively. The P value was calculated with the Spearman correlation analysis. Figure S10: correlation between THEM6 and 122 immunomodulators in Xiangya cohort. The color and the values indicate the Spearman correlation coefficient. Figures S11–S17: correlations between THEM6 and the tumor-associated immune cells calculated with the TIME algorithm, CIBERSORT-ABS algorithm, QuanTIseq algorithm, xCell algorithm, MCP-counter algorithm, TIP algorithm, and EPIC algorithm, respectively, in the Xiangya cohort. The P value was calculated with the Spea [file 7147279.f1.zip › Figure S13.png]

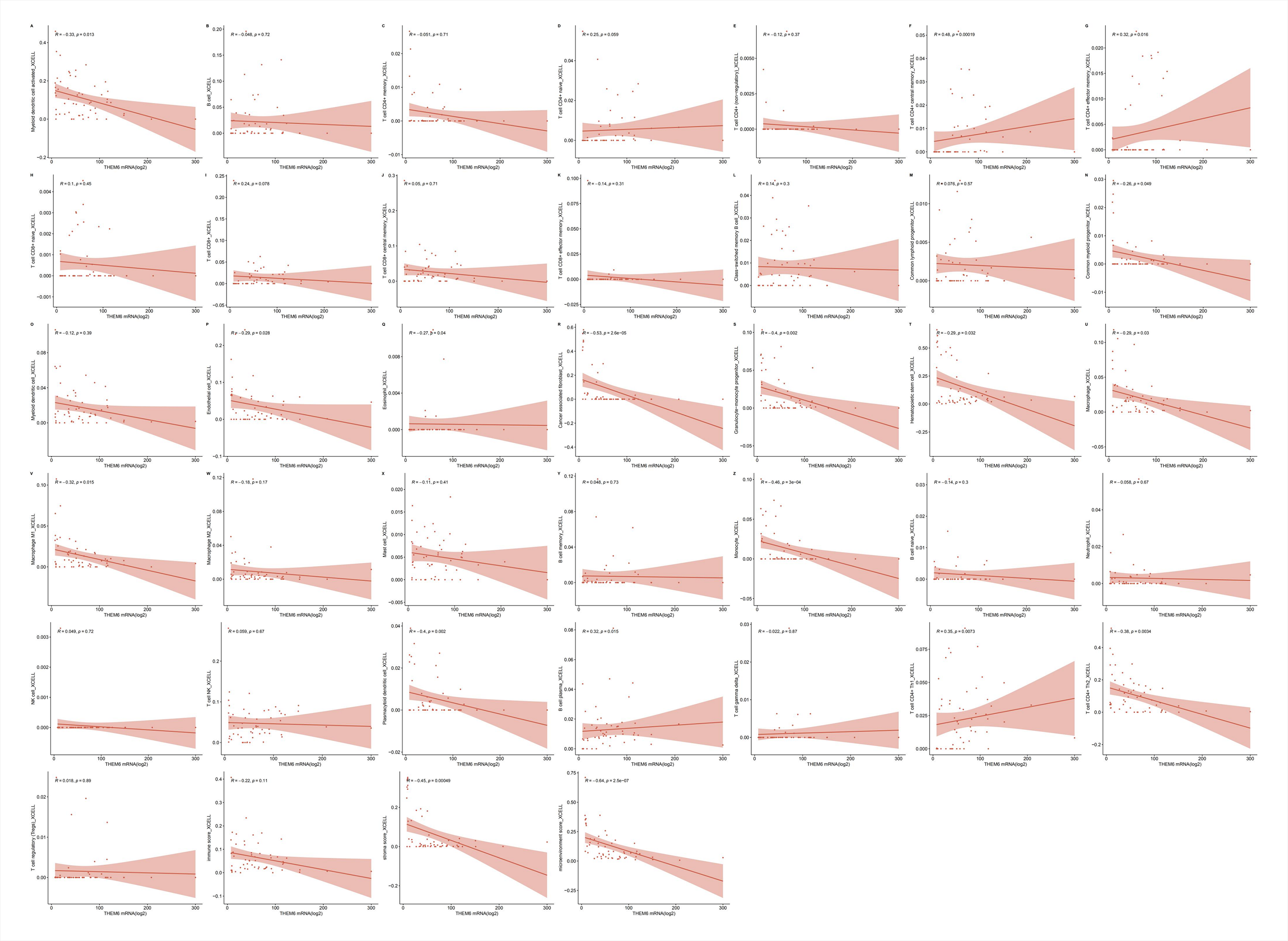

Supplement: Supplementary Materials — Figure S1: the expression pattern and prognostic analysis for overall survival of THEM6 in pan-cancers. (A, B) The expression pattern of THEM6 of pan-cancers in TCGA and GTEx. The asterisks indicated a significant statistical P value calculated with the Mann–Whitney U test (∗P < 0.05; ∗∗P < 0.01; ∗∗∗P < 0.001). (C) The prognostic analyses of THEM6 in pan-cancers using a univariate Cox regression model. Hazard ratio > 1 indicated a risk factor and hazard ratio < 1 represented a protective factor. (D) The prognostic analyses of THEM6 in pan-cancers using the Kaplan-Meier method and log-rank test. Only cancers in which THEM6 was a significant prognostic biomarker were shown. Figure S2: the correlation of THEM6 mRNA expression and immune score in pan-cancers. The P value was calculated by estimating the R page. Figure S3: the correlation of THEM6 mRNA expression and stromal score in pan-cancers. The P value was calculated by estimating the R page. Figure S4: immunological correlation of THEM6 in pan-cancers. Three cancers with the most significant differences in inflammatory cell infiltration in the pan-cancer were identified (including BLCA, BRCA, and LGG). Using the TIMER algorithm, P value calculated with the Mann–Whitney U test. Figures S5–S9: correlations between THEM6 and the tumor-associated immune cells calculated with the QuanTIseq algorithm, CIBERSORT-ABS algorithm, TISIDB algorithm, TIMER algorithm, and TIP algorithm, respectively. The P value was calculated with the Spearman correlation analysis. Figure S10: correlation between THEM6 and 122 immunomodulators in Xiangya cohort. The color and the values indicate the Spearman correlation coefficient. Figures S11–S17: correlations between THEM6 and the tumor-associated immune cells calculated with the TIME algorithm, CIBERSORT-ABS algorithm, QuanTIseq algorithm, xCell algorithm, MCP-counter algorithm, TIP algorithm, and EPIC algorithm, respectively, in the Xiangya cohort. The P value was calculated with the Spea [file 7147279.f1.zip › Figure S14.png]

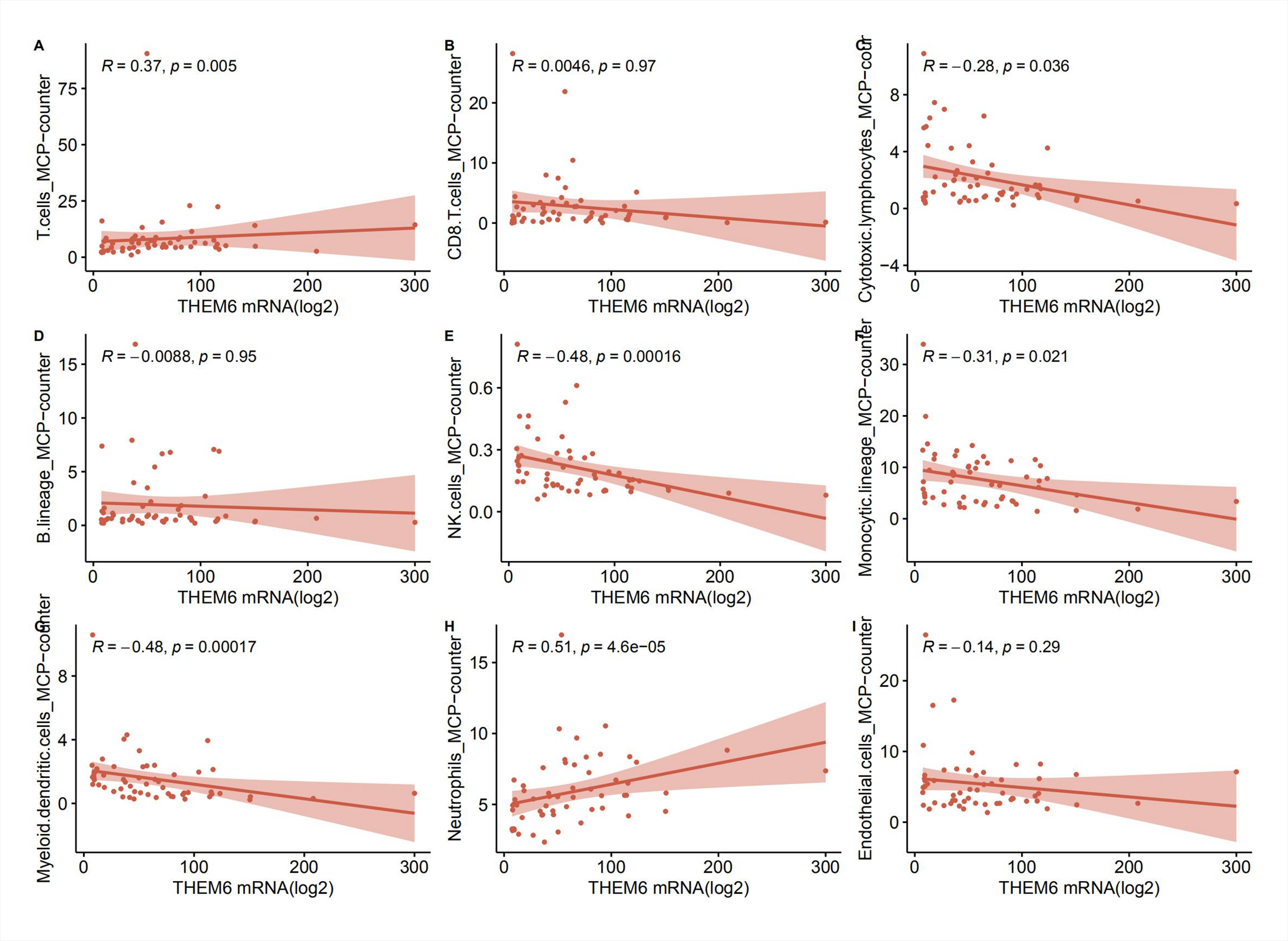

Supplement: Supplementary Materials — Figure S1: the expression pattern and prognostic analysis for overall survival of THEM6 in pan-cancers. (A, B) The expression pattern of THEM6 of pan-cancers in TCGA and GTEx. The asterisks indicated a significant statistical P value calculated with the Mann–Whitney U test (∗P < 0.05; ∗∗P < 0.01; ∗∗∗P < 0.001). (C) The prognostic analyses of THEM6 in pan-cancers using a univariate Cox regression model. Hazard ratio > 1 indicated a risk factor and hazard ratio < 1 represented a protective factor. (D) The prognostic analyses of THEM6 in pan-cancers using the Kaplan-Meier method and log-rank test. Only cancers in which THEM6 was a significant prognostic biomarker were shown. Figure S2: the correlation of THEM6 mRNA expression and immune score in pan-cancers. The P value was calculated by estimating the R page. Figure S3: the correlation of THEM6 mRNA expression and stromal score in pan-cancers. The P value was calculated by estimating the R page. Figure S4: immunological correlation of THEM6 in pan-cancers. Three cancers with the most significant differences in inflammatory cell infiltration in the pan-cancer were identified (including BLCA, BRCA, and LGG). Using the TIMER algorithm, P value calculated with the Mann–Whitney U test. Figures S5–S9: correlations between THEM6 and the tumor-associated immune cells calculated with the QuanTIseq algorithm, CIBERSORT-ABS algorithm, TISIDB algorithm, TIMER algorithm, and TIP algorithm, respectively. The P value was calculated with the Spearman correlation analysis. Figure S10: correlation between THEM6 and 122 immunomodulators in Xiangya cohort. The color and the values indicate the Spearman correlation coefficient. Figures S11–S17: correlations between THEM6 and the tumor-associated immune cells calculated with the TIME algorithm, CIBERSORT-ABS algorithm, QuanTIseq algorithm, xCell algorithm, MCP-counter algorithm, TIP algorithm, and EPIC algorithm, respectively, in the Xiangya cohort. The P value was calculated with the Spea [file 7147279.f1.zip › Figure S15.png]

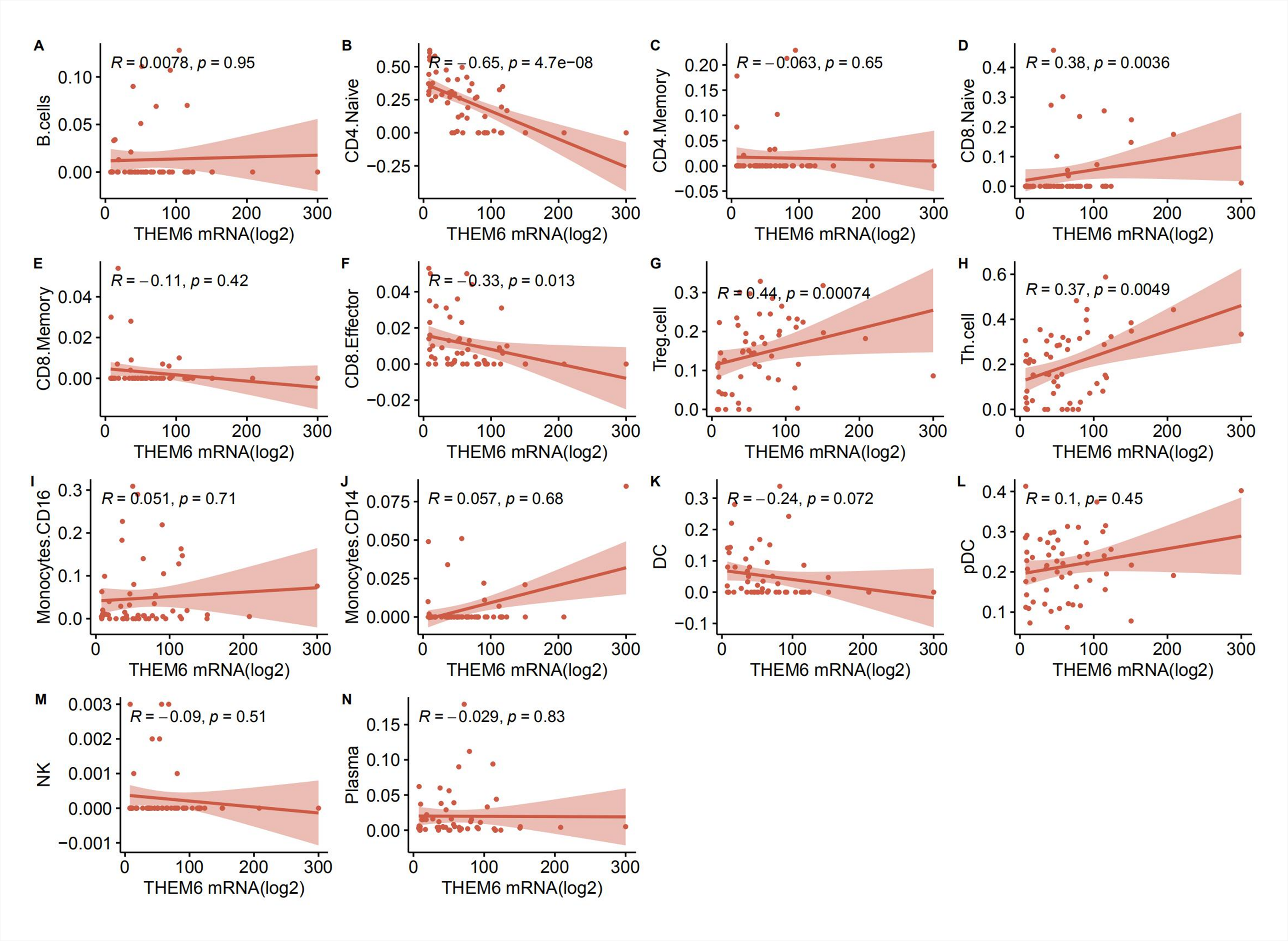

Supplement: Supplementary Materials — Figure S1: the expression pattern and prognostic analysis for overall survival of THEM6 in pan-cancers. (A, B) The expression pattern of THEM6 of pan-cancers in TCGA and GTEx. The asterisks indicated a significant statistical P value calculated with the Mann–Whitney U test (∗P < 0.05; ∗∗P < 0.01; ∗∗∗P < 0.001). (C) The prognostic analyses of THEM6 in pan-cancers using a univariate Cox regression model. Hazard ratio > 1 indicated a risk factor and hazard ratio < 1 represented a protective factor. (D) The prognostic analyses of THEM6 in pan-cancers using the Kaplan-Meier method and log-rank test. Only cancers in which THEM6 was a significant prognostic biomarker were shown. Figure S2: the correlation of THEM6 mRNA expression and immune score in pan-cancers. The P value was calculated by estimating the R page. Figure S3: the correlation of THEM6 mRNA expression and stromal score in pan-cancers. The P value was calculated by estimating the R page. Figure S4: immunological correlation of THEM6 in pan-cancers. Three cancers with the most significant differences in inflammatory cell infiltration in the pan-cancer were identified (including BLCA, BRCA, and LGG). Using the TIMER algorithm, P value calculated with the Mann–Whitney U test. Figures S5–S9: correlations between THEM6 and the tumor-associated immune cells calculated with the QuanTIseq algorithm, CIBERSORT-ABS algorithm, TISIDB algorithm, TIMER algorithm, and TIP algorithm, respectively. The P value was calculated with the Spearman correlation analysis. Figure S10: correlation between THEM6 and 122 immunomodulators in Xiangya cohort. The color and the values indicate the Spearman correlation coefficient. Figures S11–S17: correlations between THEM6 and the tumor-associated immune cells calculated with the TIME algorithm, CIBERSORT-ABS algorithm, QuanTIseq algorithm, xCell algorithm, MCP-counter algorithm, TIP algorithm, and EPIC algorithm, respectively, in the Xiangya cohort. The P value was calculated with the Spea [file 7147279.f1.zip › Figure S16.png]

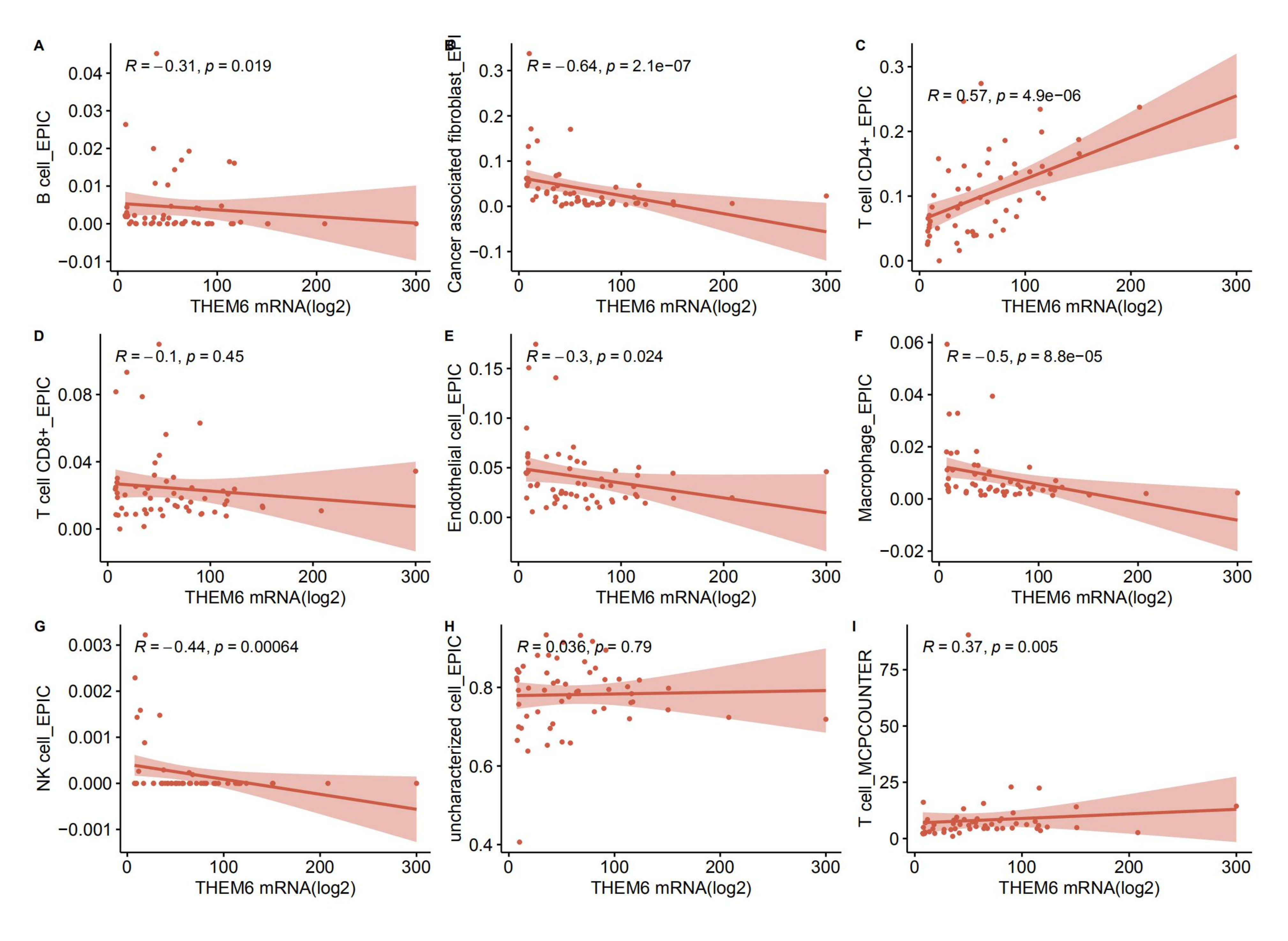

Supplement: Supplementary Materials — Figure S1: the expression pattern and prognostic analysis for overall survival of THEM6 in pan-cancers. (A, B) The expression pattern of THEM6 of pan-cancers in TCGA and GTEx. The asterisks indicated a significant statistical P value calculated with the Mann–Whitney U test (∗P < 0.05; ∗∗P < 0.01; ∗∗∗P < 0.001). (C) The prognostic analyses of THEM6 in pan-cancers using a univariate Cox regression model. Hazard ratio > 1 indicated a risk factor and hazard ratio < 1 represented a protective factor. (D) The prognostic analyses of THEM6 in pan-cancers using the Kaplan-Meier method and log-rank test. Only cancers in which THEM6 was a significant prognostic biomarker were shown. Figure S2: the correlation of THEM6 mRNA expression and immune score in pan-cancers. The P value was calculated by estimating the R page. Figure S3: the correlation of THEM6 mRNA expression and stromal score in pan-cancers. The P value was calculated by estimating the R page. Figure S4: immunological correlation of THEM6 in pan-cancers. Three cancers with the most significant differences in inflammatory cell infiltration in the pan-cancer were identified (including BLCA, BRCA, and LGG). Using the TIMER algorithm, P value calculated with the Mann–Whitney U test. Figures S5–S9: correlations between THEM6 and the tumor-associated immune cells calculated with the QuanTIseq algorithm, CIBERSORT-ABS algorithm, TISIDB algorithm, TIMER algorithm, and TIP algorithm, respectively. The P value was calculated with the Spearman correlation analysis. Figure S10: correlation between THEM6 and 122 immunomodulators in Xiangya cohort. The color and the values indicate the Spearman correlation coefficient. Figures S11–S17: correlations between THEM6 and the tumor-associated immune cells calculated with the TIME algorithm, CIBERSORT-ABS algorithm, QuanTIseq algorithm, xCell algorithm, MCP-counter algorithm, TIP algorithm, and EPIC algorithm, respectively, in the Xiangya cohort. The P value was calculated with the Spea [file 7147279.f1.zip › Figure S17.png]

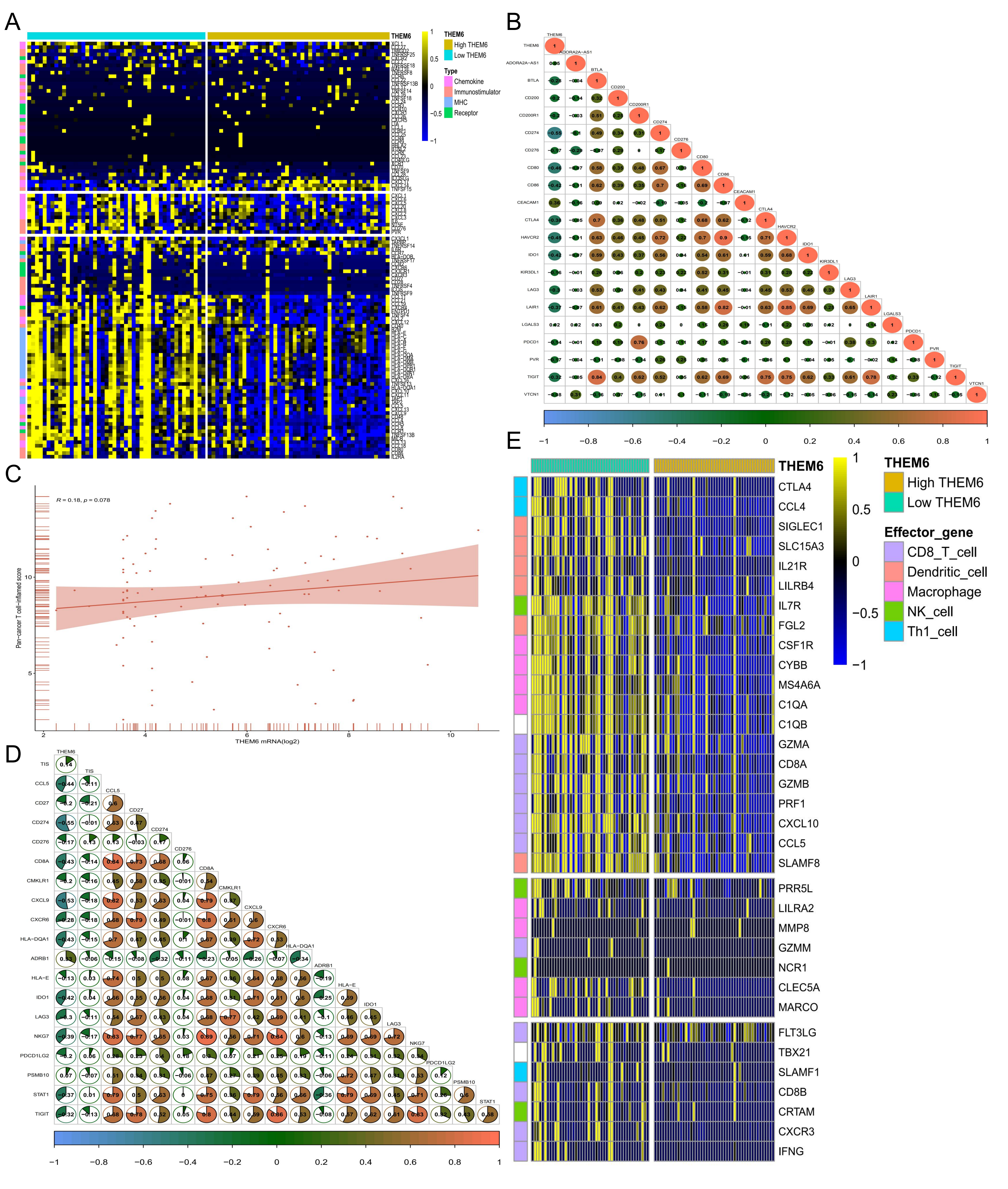

Supplement: Supplementary Materials — Figure S1: the expression pattern and prognostic analysis for overall survival of THEM6 in pan-cancers. (A, B) The expression pattern of THEM6 of pan-cancers in TCGA and GTEx. The asterisks indicated a significant statistical P value calculated with the Mann–Whitney U test (∗P < 0.05; ∗∗P < 0.01; ∗∗∗P < 0.001). (C) The prognostic analyses of THEM6 in pan-cancers using a univariate Cox regression model. Hazard ratio > 1 indicated a risk factor and hazard ratio < 1 represented a protective factor. (D) The prognostic analyses of THEM6 in pan-cancers using the Kaplan-Meier method and log-rank test. Only cancers in which THEM6 was a significant prognostic biomarker were shown. Figure S2: the correlation of THEM6 mRNA expression and immune score in pan-cancers. The P value was calculated by estimating the R page. Figure S3: the correlation of THEM6 mRNA expression and stromal score in pan-cancers. The P value was calculated by estimating the R page. Figure S4: immunological correlation of THEM6 in pan-cancers. Three cancers with the most significant differences in inflammatory cell infiltration in the pan-cancer were identified (including BLCA, BRCA, and LGG). Using the TIMER algorithm, P value calculated with the Mann–Whitney U test. Figures S5–S9: correlations between THEM6 and the tumor-associated immune cells calculated with the QuanTIseq algorithm, CIBERSORT-ABS algorithm, TISIDB algorithm, TIMER algorithm, and TIP algorithm, respectively. The P value was calculated with the Spearman correlation analysis. Figure S10: correlation between THEM6 and 122 immunomodulators in Xiangya cohort. The color and the values indicate the Spearman correlation coefficient. Figures S11–S17: correlations between THEM6 and the tumor-associated immune cells calculated with the TIME algorithm, CIBERSORT-ABS algorithm, QuanTIseq algorithm, xCell algorithm, MCP-counter algorithm, TIP algorithm, and EPIC algorithm, respectively, in the Xiangya cohort. The P value was calculated with the Spea [file 7147279.f1.zip › Figure S18.png]

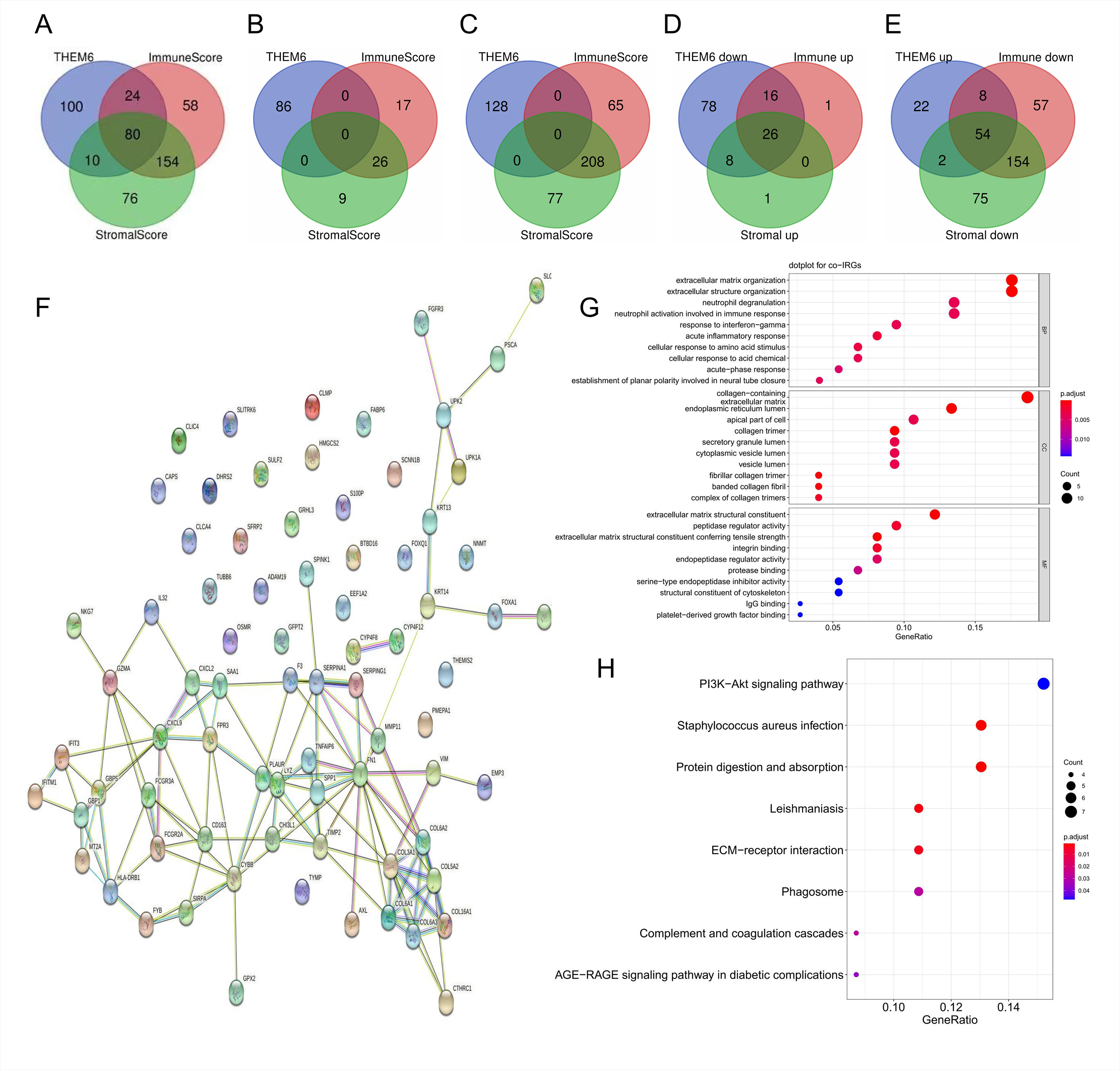

Supplement: Supplementary Materials — Figure S1: the expression pattern and prognostic analysis for overall survival of THEM6 in pan-cancers. (A, B) The expression pattern of THEM6 of pan-cancers in TCGA and GTEx. The asterisks indicated a significant statistical P value calculated with the Mann–Whitney U test (∗P < 0.05; ∗∗P < 0.01; ∗∗∗P < 0.001). (C) The prognostic analyses of THEM6 in pan-cancers using a univariate Cox regression model. Hazard ratio > 1 indicated a risk factor and hazard ratio < 1 represented a protective factor. (D) The prognostic analyses of THEM6 in pan-cancers using the Kaplan-Meier method and log-rank test. Only cancers in which THEM6 was a significant prognostic biomarker were shown. Figure S2: the correlation of THEM6 mRNA expression and immune score in pan-cancers. The P value was calculated by estimating the R page. Figure S3: the correlation of THEM6 mRNA expression and stromal score in pan-cancers. The P value was calculated by estimating the R page. Figure S4: immunological correlation of THEM6 in pan-cancers. Three cancers with the most significant differences in inflammatory cell infiltration in the pan-cancer were identified (including BLCA, BRCA, and LGG). Using the TIMER algorithm, P value calculated with the Mann–Whitney U test. Figures S5–S9: correlations between THEM6 and the tumor-associated immune cells calculated with the QuanTIseq algorithm, CIBERSORT-ABS algorithm, TISIDB algorithm, TIMER algorithm, and TIP algorithm, respectively. The P value was calculated with the Spearman correlation analysis. Figure S10: correlation between THEM6 and 122 immunomodulators in Xiangya cohort. The color and the values indicate the Spearman correlation coefficient. Figures S11–S17: correlations between THEM6 and the tumor-associated immune cells calculated with the TIME algorithm, CIBERSORT-ABS algorithm, QuanTIseq algorithm, xCell algorithm, MCP-counter algorithm, TIP algorithm, and EPIC algorithm, respectively, in the Xiangya cohort. The P value was calculated with the Spea [file 7147279.f1.zip › Figure S19.png]

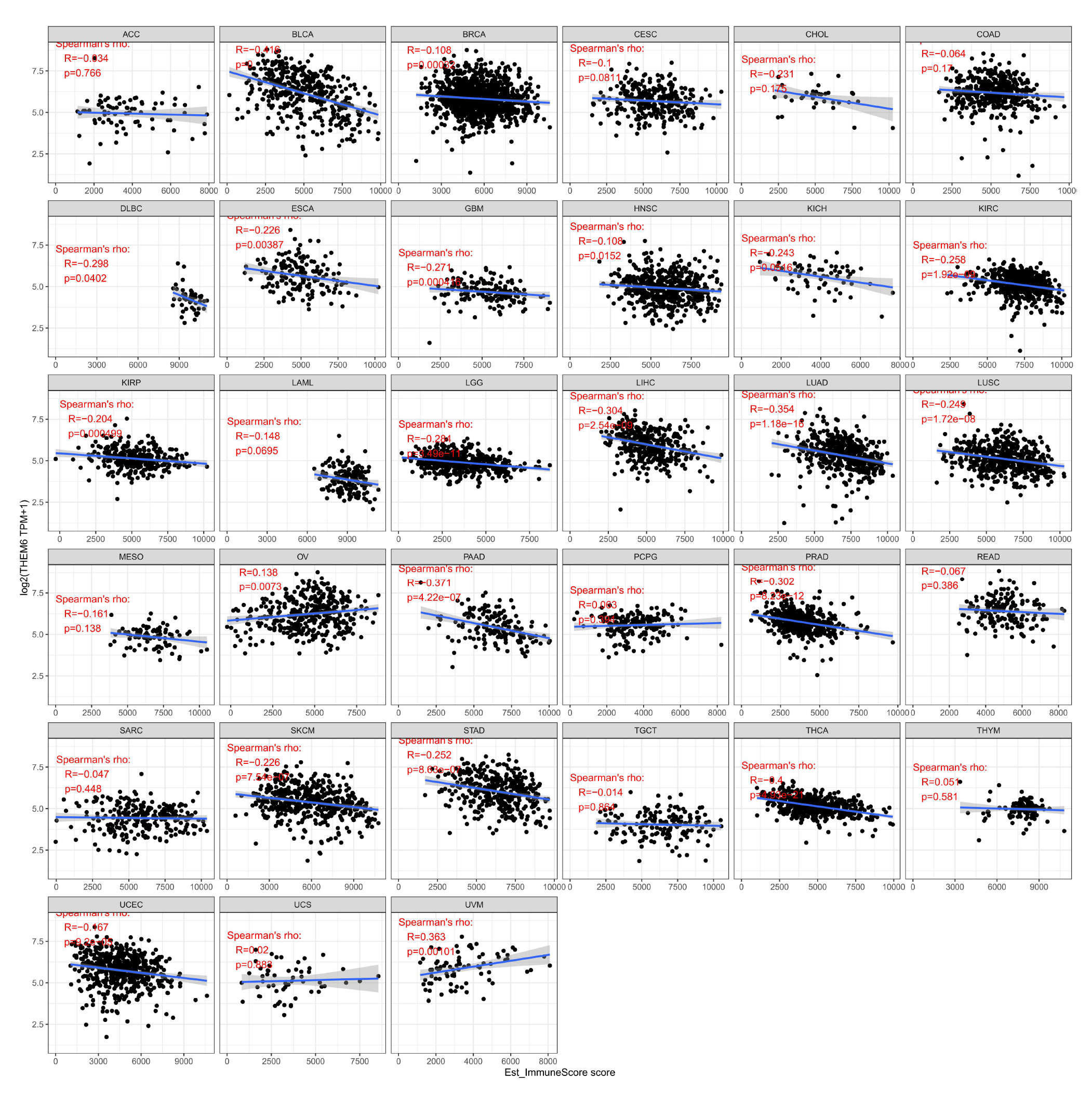

Supplement: Supplementary Materials — Figure S1: the expression pattern and prognostic analysis for overall survival of THEM6 in pan-cancers. (A, B) The expression pattern of THEM6 of pan-cancers in TCGA and GTEx. The asterisks indicated a significant statistical P value calculated with the Mann–Whitney U test (∗P < 0.05; ∗∗P < 0.01; ∗∗∗P < 0.001). (C) The prognostic analyses of THEM6 in pan-cancers using a univariate Cox regression model. Hazard ratio > 1 indicated a risk factor and hazard ratio < 1 represented a protective factor. (D) The prognostic analyses of THEM6 in pan-cancers using the Kaplan-Meier method and log-rank test. Only cancers in which THEM6 was a significant prognostic biomarker were shown. Figure S2: the correlation of THEM6 mRNA expression and immune score in pan-cancers. The P value was calculated by estimating the R page. Figure S3: the correlation of THEM6 mRNA expression and stromal score in pan-cancers. The P value was calculated by estimating the R page. Figure S4: immunological correlation of THEM6 in pan-cancers. Three cancers with the most significant differences in inflammatory cell infiltration in the pan-cancer were identified (including BLCA, BRCA, and LGG). Using the TIMER algorithm, P value calculated with the Mann–Whitney U test. Figures S5–S9: correlations between THEM6 and the tumor-associated immune cells calculated with the QuanTIseq algorithm, CIBERSORT-ABS algorithm, TISIDB algorithm, TIMER algorithm, and TIP algorithm, respectively. The P value was calculated with the Spearman correlation analysis. Figure S10: correlation between THEM6 and 122 immunomodulators in Xiangya cohort. The color and the values indicate the Spearman correlation coefficient. Figures S11–S17: correlations between THEM6 and the tumor-associated immune cells calculated with the TIME algorithm, CIBERSORT-ABS algorithm, QuanTIseq algorithm, xCell algorithm, MCP-counter algorithm, TIP algorithm, and EPIC algorithm, respectively, in the Xiangya cohort. The P value was calculated with the Spea [file 7147279.f1.zip › Figure S2.png]

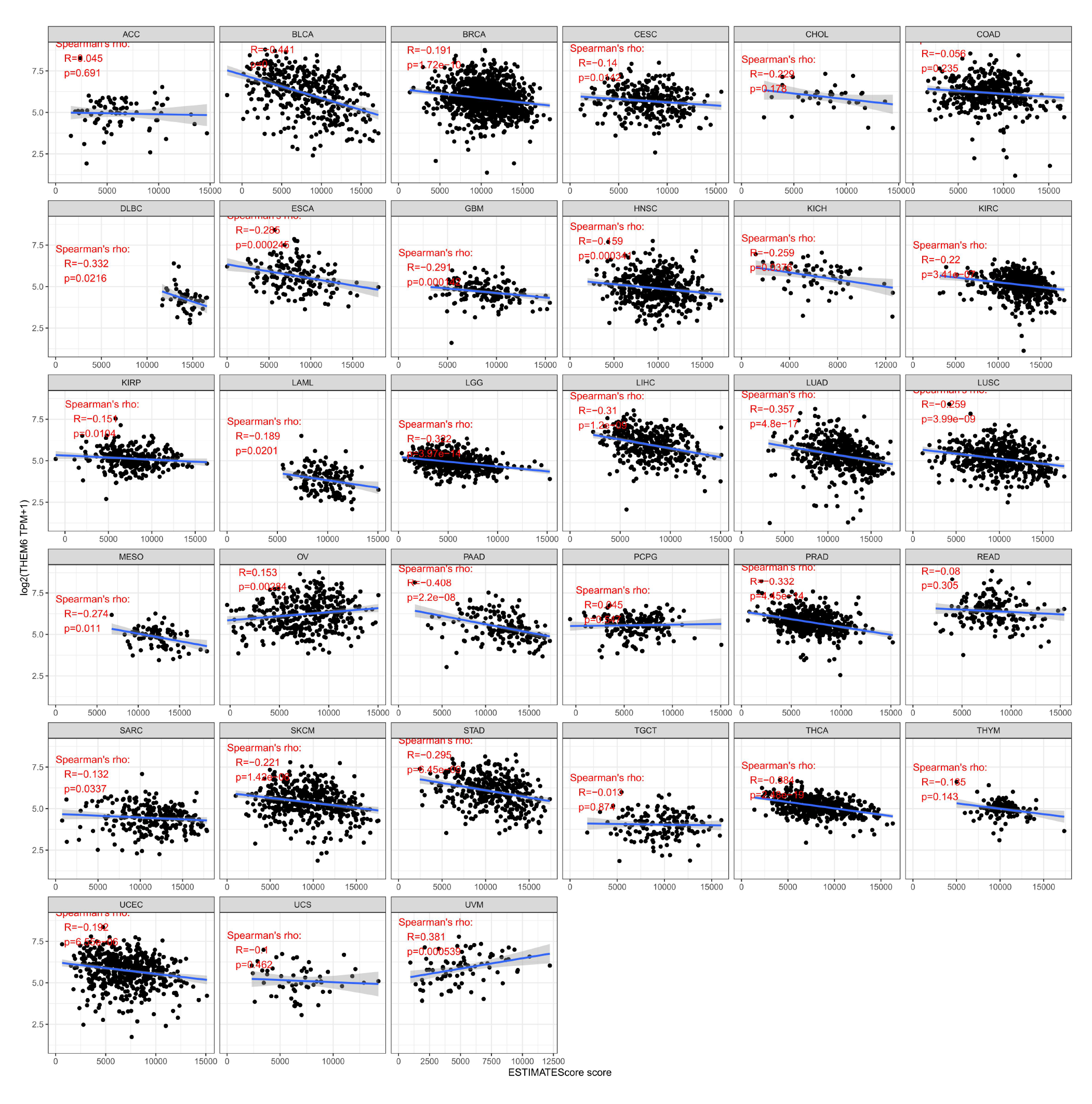

Supplement: Supplementary Materials — Figure S1: the expression pattern and prognostic analysis for overall survival of THEM6 in pan-cancers. (A, B) The expression pattern of THEM6 of pan-cancers in TCGA and GTEx. The asterisks indicated a significant statistical P value calculated with the Mann–Whitney U test (∗P < 0.05; ∗∗P < 0.01; ∗∗∗P < 0.001). (C) The prognostic analyses of THEM6 in pan-cancers using a univariate Cox regression model. Hazard ratio > 1 indicated a risk factor and hazard ratio < 1 represented a protective factor. (D) The prognostic analyses of THEM6 in pan-cancers using the Kaplan-Meier method and log-rank test. Only cancers in which THEM6 was a significant prognostic biomarker were shown. Figure S2: the correlation of THEM6 mRNA expression and immune score in pan-cancers. The P value was calculated by estimating the R page. Figure S3: the correlation of THEM6 mRNA expression and stromal score in pan-cancers. The P value was calculated by estimating the R page. Figure S4: immunological correlation of THEM6 in pan-cancers. Three cancers with the most significant differences in inflammatory cell infiltration in the pan-cancer were identified (including BLCA, BRCA, and LGG). Using the TIMER algorithm, P value calculated with the Mann–Whitney U test. Figures S5–S9: correlations between THEM6 and the tumor-associated immune cells calculated with the QuanTIseq algorithm, CIBERSORT-ABS algorithm, TISIDB algorithm, TIMER algorithm, and TIP algorithm, respectively. The P value was calculated with the Spearman correlation analysis. Figure S10: correlation between THEM6 and 122 immunomodulators in Xiangya cohort. The color and the values indicate the Spearman correlation coefficient. Figures S11–S17: correlations between THEM6 and the tumor-associated immune cells calculated with the TIME algorithm, CIBERSORT-ABS algorithm, QuanTIseq algorithm, xCell algorithm, MCP-counter algorithm, TIP algorithm, and EPIC algorithm, respectively, in the Xiangya cohort. The P value was calculated with the Spea [file 7147279.f1.zip › Figure S3.png]

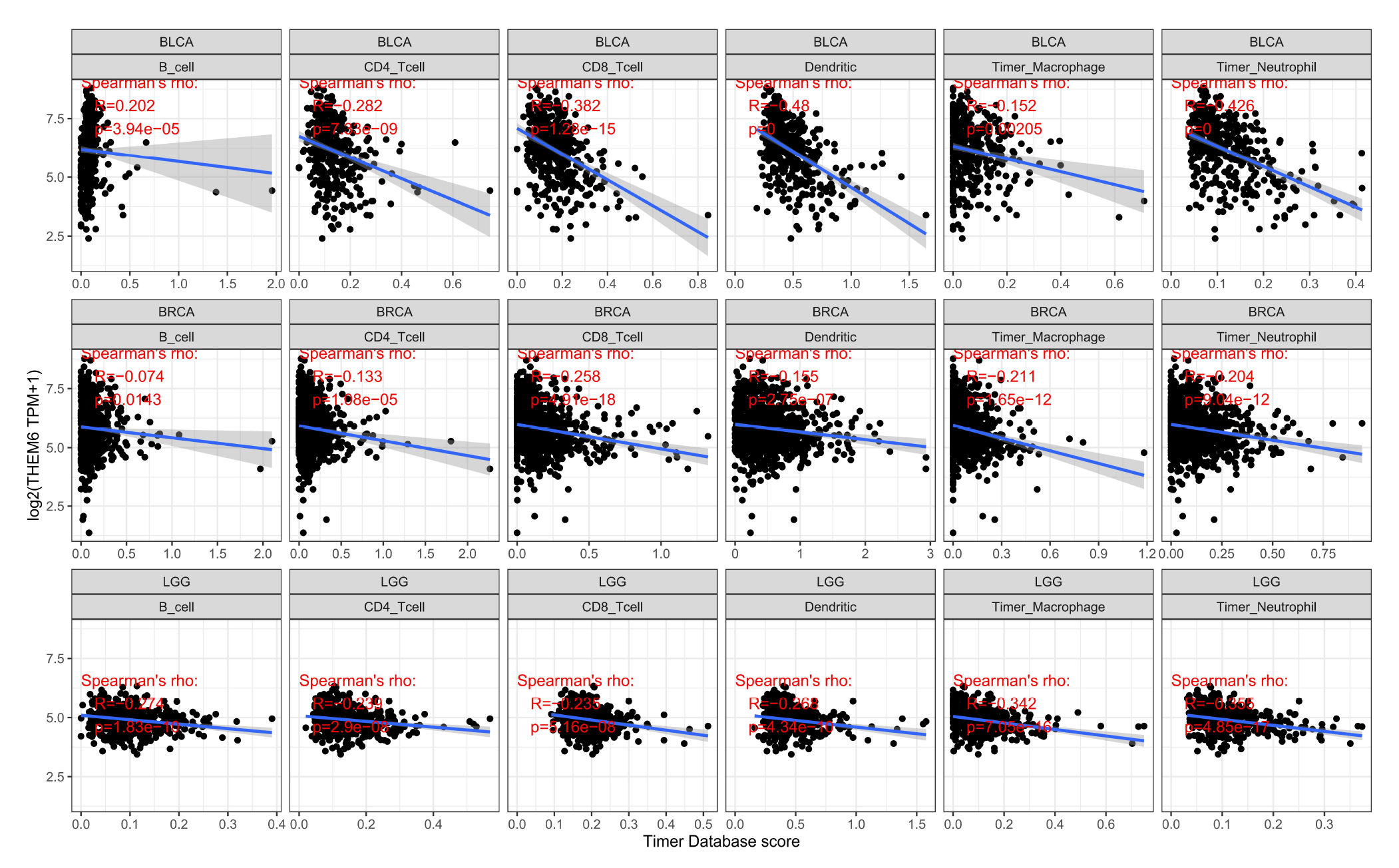

Supplement: Supplementary Materials — Figure S1: the expression pattern and prognostic analysis for overall survival of THEM6 in pan-cancers. (A, B) The expression pattern of THEM6 of pan-cancers in TCGA and GTEx. The asterisks indicated a significant statistical P value calculated with the Mann–Whitney U test (∗P < 0.05; ∗∗P < 0.01; ∗∗∗P < 0.001). (C) The prognostic analyses of THEM6 in pan-cancers using a univariate Cox regression model. Hazard ratio > 1 indicated a risk factor and hazard ratio < 1 represented a protective factor. (D) The prognostic analyses of THEM6 in pan-cancers using the Kaplan-Meier method and log-rank test. Only cancers in which THEM6 was a significant prognostic biomarker were shown. Figure S2: the correlation of THEM6 mRNA expression and immune score in pan-cancers. The P value was calculated by estimating the R page. Figure S3: the correlation of THEM6 mRNA expression and stromal score in pan-cancers. The P value was calculated by estimating the R page. Figure S4: immunological correlation of THEM6 in pan-cancers. Three cancers with the most significant differences in inflammatory cell infiltration in the pan-cancer were identified (including BLCA, BRCA, and LGG). Using the TIMER algorithm, P value calculated with the Mann–Whitney U test. Figures S5–S9: correlations between THEM6 and the tumor-associated immune cells calculated with the QuanTIseq algorithm, CIBERSORT-ABS algorithm, TISIDB algorithm, TIMER algorithm, and TIP algorithm, respectively. The P value was calculated with the Spearman correlation analysis. Figure S10: correlation between THEM6 and 122 immunomodulators in Xiangya cohort. The color and the values indicate the Spearman correlation coefficient. Figures S11–S17: correlations between THEM6 and the tumor-associated immune cells calculated with the TIME algorithm, CIBERSORT-ABS algorithm, QuanTIseq algorithm, xCell algorithm, MCP-counter algorithm, TIP algorithm, and EPIC algorithm, respectively, in the Xiangya cohort. The P value was calculated with the Spea [file 7147279.f1.zip › Figure S4.png]

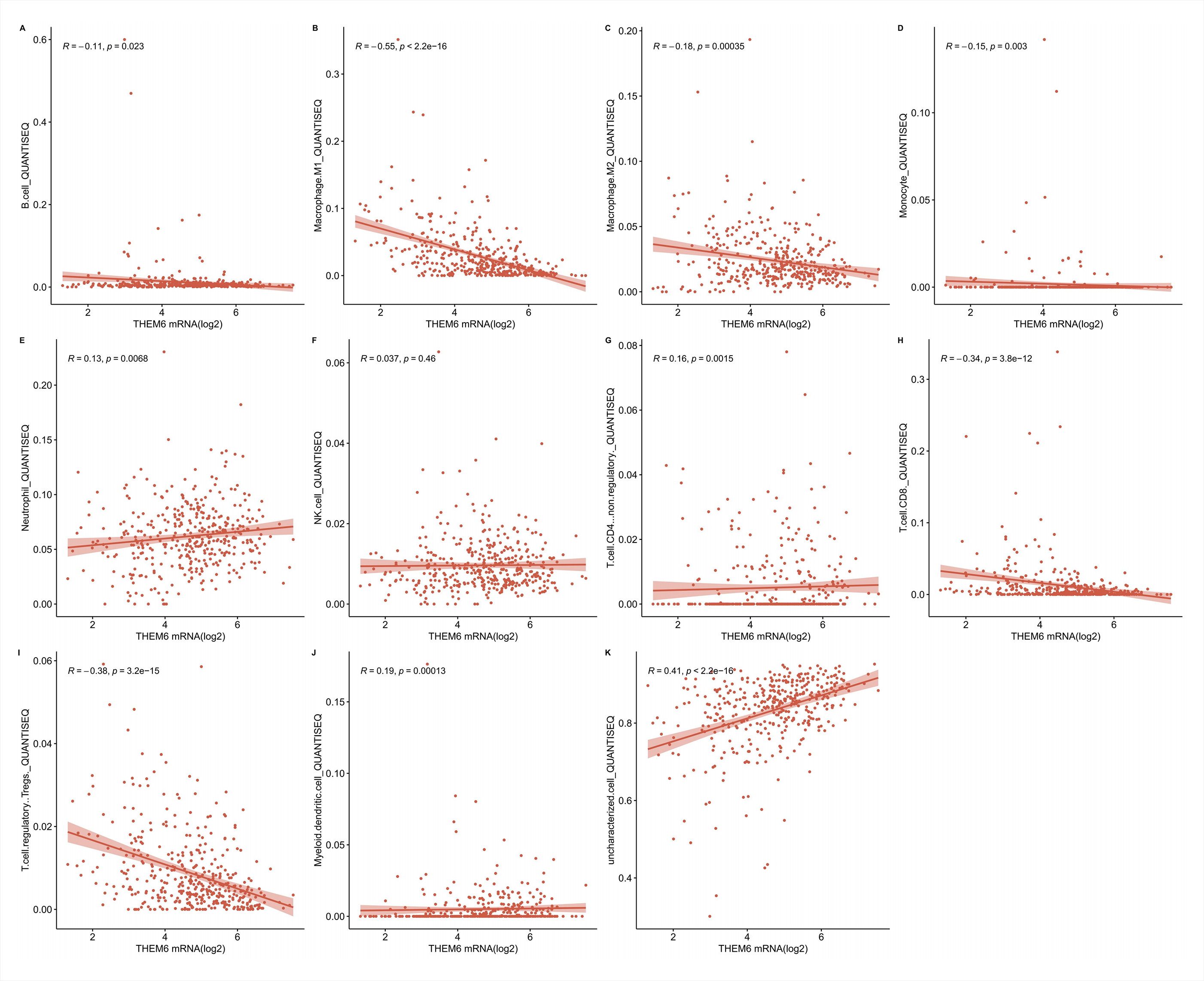

Supplement: Supplementary Materials — Figure S1: the expression pattern and prognostic analysis for overall survival of THEM6 in pan-cancers. (A, B) The expression pattern of THEM6 of pan-cancers in TCGA and GTEx. The asterisks indicated a significant statistical P value calculated with the Mann–Whitney U test (∗P < 0.05; ∗∗P < 0.01; ∗∗∗P < 0.001). (C) The prognostic analyses of THEM6 in pan-cancers using a univariate Cox regression model. Hazard ratio > 1 indicated a risk factor and hazard ratio < 1 represented a protective factor. (D) The prognostic analyses of THEM6 in pan-cancers using the Kaplan-Meier method and log-rank test. Only cancers in which THEM6 was a significant prognostic biomarker were shown. Figure S2: the correlation of THEM6 mRNA expression and immune score in pan-cancers. The P value was calculated by estimating the R page. Figure S3: the correlation of THEM6 mRNA expression and stromal score in pan-cancers. The P value was calculated by estimating the R page. Figure S4: immunological correlation of THEM6 in pan-cancers. Three cancers with the most significant differences in inflammatory cell infiltration in the pan-cancer were identified (including BLCA, BRCA, and LGG). Using the TIMER algorithm, P value calculated with the Mann–Whitney U test. Figures S5–S9: correlations between THEM6 and the tumor-associated immune cells calculated with the QuanTIseq algorithm, CIBERSORT-ABS algorithm, TISIDB algorithm, TIMER algorithm, and TIP algorithm, respectively. The P value was calculated with the Spearman correlation analysis. Figure S10: correlation between THEM6 and 122 immunomodulators in Xiangya cohort. The color and the values indicate the Spearman correlation coefficient. Figures S11–S17: correlations between THEM6 and the tumor-associated immune cells calculated with the TIME algorithm, CIBERSORT-ABS algorithm, QuanTIseq algorithm, xCell algorithm, MCP-counter algorithm, TIP algorithm, and EPIC algorithm, respectively, in the Xiangya cohort. The P value was calculated with the Spea [file 7147279.f1.zip › Figure S5.png]

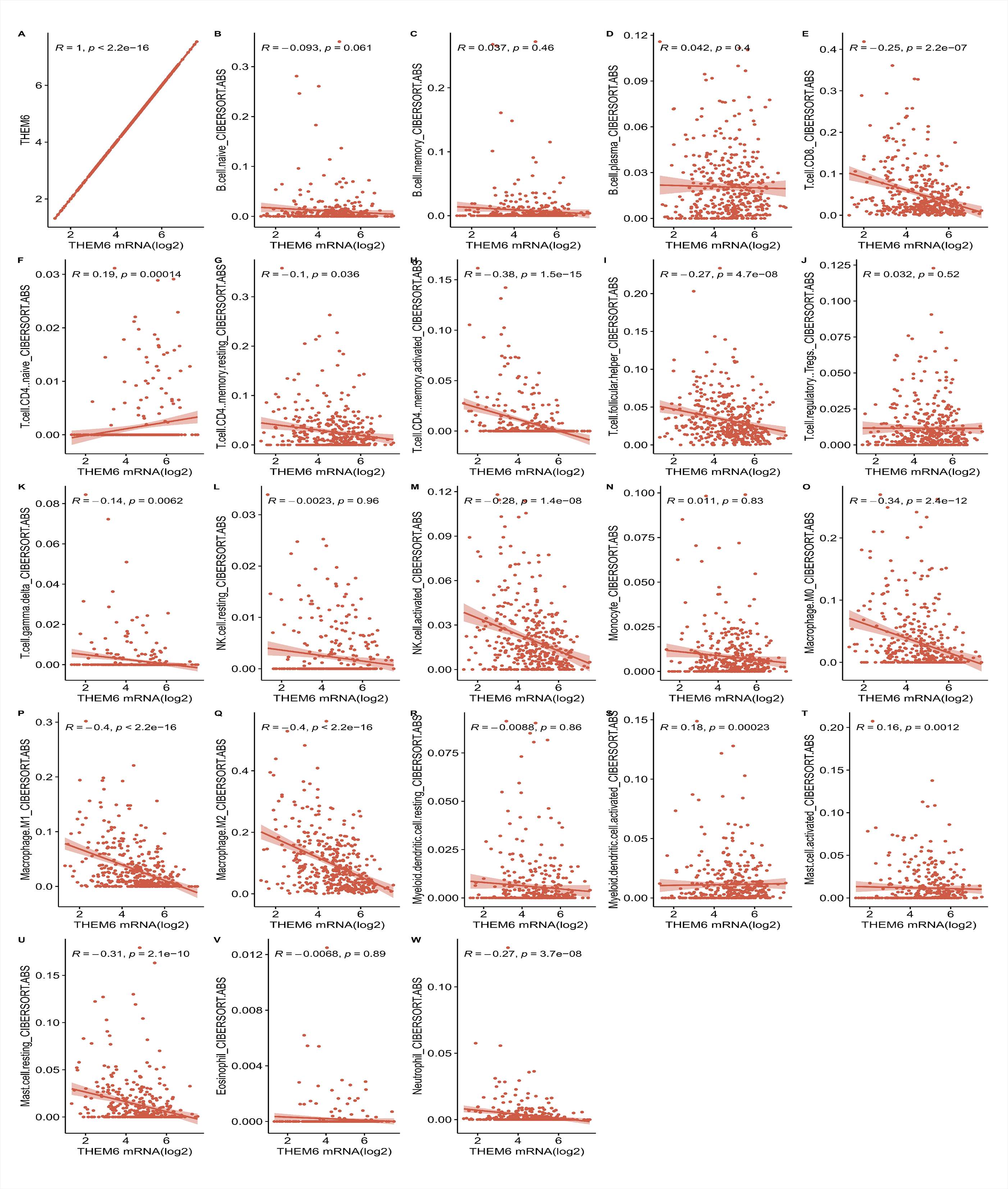

Supplement: Supplementary Materials — Figure S1: the expression pattern and prognostic analysis for overall survival of THEM6 in pan-cancers. (A, B) The expression pattern of THEM6 of pan-cancers in TCGA and GTEx. The asterisks indicated a significant statistical P value calculated with the Mann–Whitney U test (∗P < 0.05; ∗∗P < 0.01; ∗∗∗P < 0.001). (C) The prognostic analyses of THEM6 in pan-cancers using a univariate Cox regression model. Hazard ratio > 1 indicated a risk factor and hazard ratio < 1 represented a protective factor. (D) The prognostic analyses of THEM6 in pan-cancers using the Kaplan-Meier method and log-rank test. Only cancers in which THEM6 was a significant prognostic biomarker were shown. Figure S2: the correlation of THEM6 mRNA expression and immune score in pan-cancers. The P value was calculated by estimating the R page. Figure S3: the correlation of THEM6 mRNA expression and stromal score in pan-cancers. The P value was calculated by estimating the R page. Figure S4: immunological correlation of THEM6 in pan-cancers. Three cancers with the most significant differences in inflammatory cell infiltration in the pan-cancer were identified (including BLCA, BRCA, and LGG). Using the TIMER algorithm, P value calculated with the Mann–Whitney U test. Figures S5–S9: correlations between THEM6 and the tumor-associated immune cells calculated with the QuanTIseq algorithm, CIBERSORT-ABS algorithm, TISIDB algorithm, TIMER algorithm, and TIP algorithm, respectively. The P value was calculated with the Spearman correlation analysis. Figure S10: correlation between THEM6 and 122 immunomodulators in Xiangya cohort. The color and the values indicate the Spearman correlation coefficient. Figures S11–S17: correlations between THEM6 and the tumor-associated immune cells calculated with the TIME algorithm, CIBERSORT-ABS algorithm, QuanTIseq algorithm, xCell algorithm, MCP-counter algorithm, TIP algorithm, and EPIC algorithm, respectively, in the Xiangya cohort. The P value was calculated with the Spea [file 7147279.f1.zip › Figure S6.png]

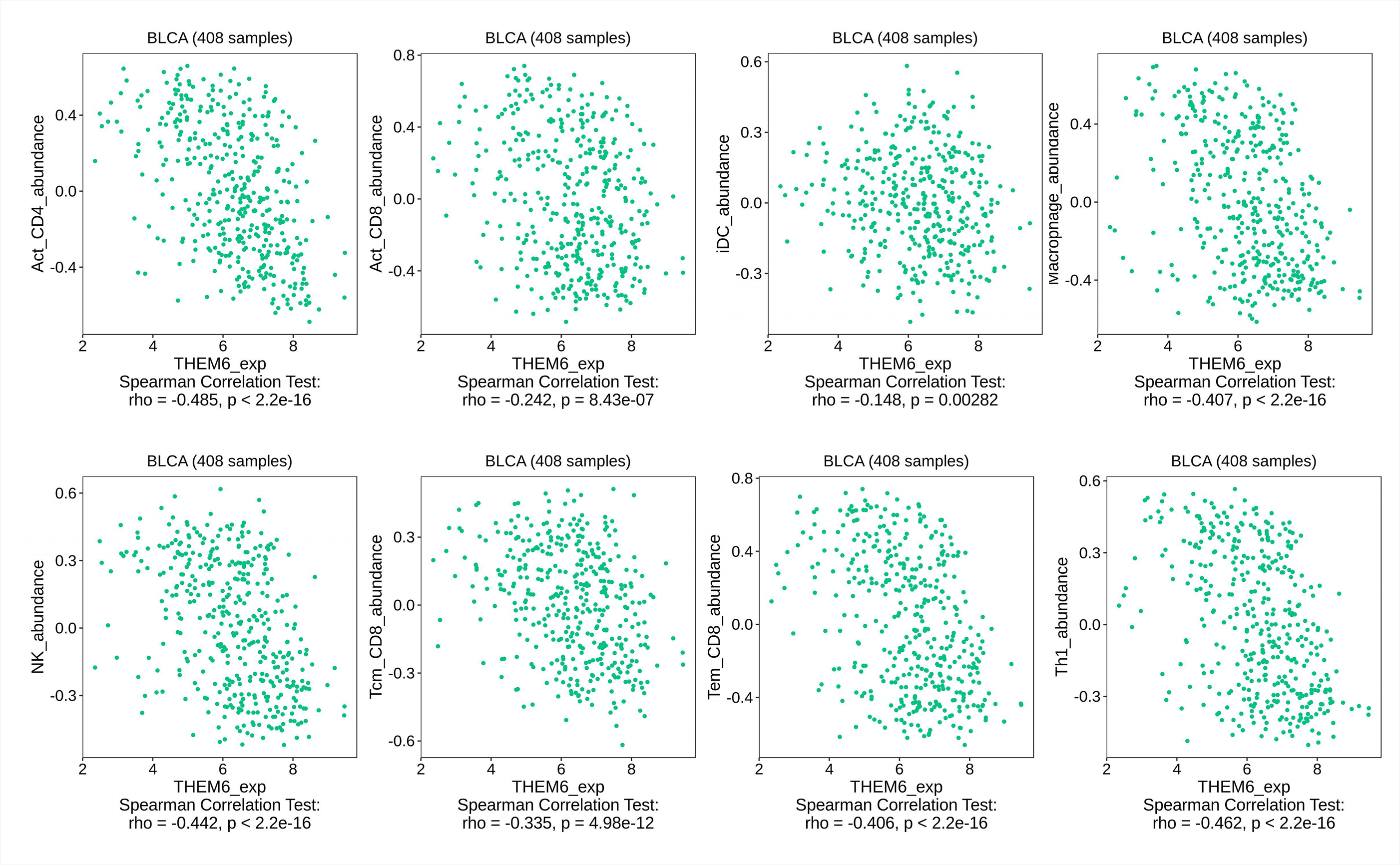

Supplement: Supplementary Materials — Figure S1: the expression pattern and prognostic analysis for overall survival of THEM6 in pan-cancers. (A, B) The expression pattern of THEM6 of pan-cancers in TCGA and GTEx. The asterisks indicated a significant statistical P value calculated with the Mann–Whitney U test (∗P < 0.05; ∗∗P < 0.01; ∗∗∗P < 0.001). (C) The prognostic analyses of THEM6 in pan-cancers using a univariate Cox regression model. Hazard ratio > 1 indicated a risk factor and hazard ratio < 1 represented a protective factor. (D) The prognostic analyses of THEM6 in pan-cancers using the Kaplan-Meier method and log-rank test. Only cancers in which THEM6 was a significant prognostic biomarker were shown. Figure S2: the correlation of THEM6 mRNA expression and immune score in pan-cancers. The P value was calculated by estimating the R page. Figure S3: the correlation of THEM6 mRNA expression and stromal score in pan-cancers. The P value was calculated by estimating the R page. Figure S4: immunological correlation of THEM6 in pan-cancers. Three cancers with the most significant differences in inflammatory cell infiltration in the pan-cancer were identified (including BLCA, BRCA, and LGG). Using the TIMER algorithm, P value calculated with the Mann–Whitney U test. Figures S5–S9: correlations between THEM6 and the tumor-associated immune cells calculated with the QuanTIseq algorithm, CIBERSORT-ABS algorithm, TISIDB algorithm, TIMER algorithm, and TIP algorithm, respectively. The P value was calculated with the Spearman correlation analysis. Figure S10: correlation between THEM6 and 122 immunomodulators in Xiangya cohort. The color and the values indicate the Spearman correlation coefficient. Figures S11–S17: correlations between THEM6 and the tumor-associated immune cells calculated with the TIME algorithm, CIBERSORT-ABS algorithm, QuanTIseq algorithm, xCell algorithm, MCP-counter algorithm, TIP algorithm, and EPIC algorithm, respectively, in the Xiangya cohort. The P value was calculated with the Spea [file 7147279.f1.zip › Figure S7.png]

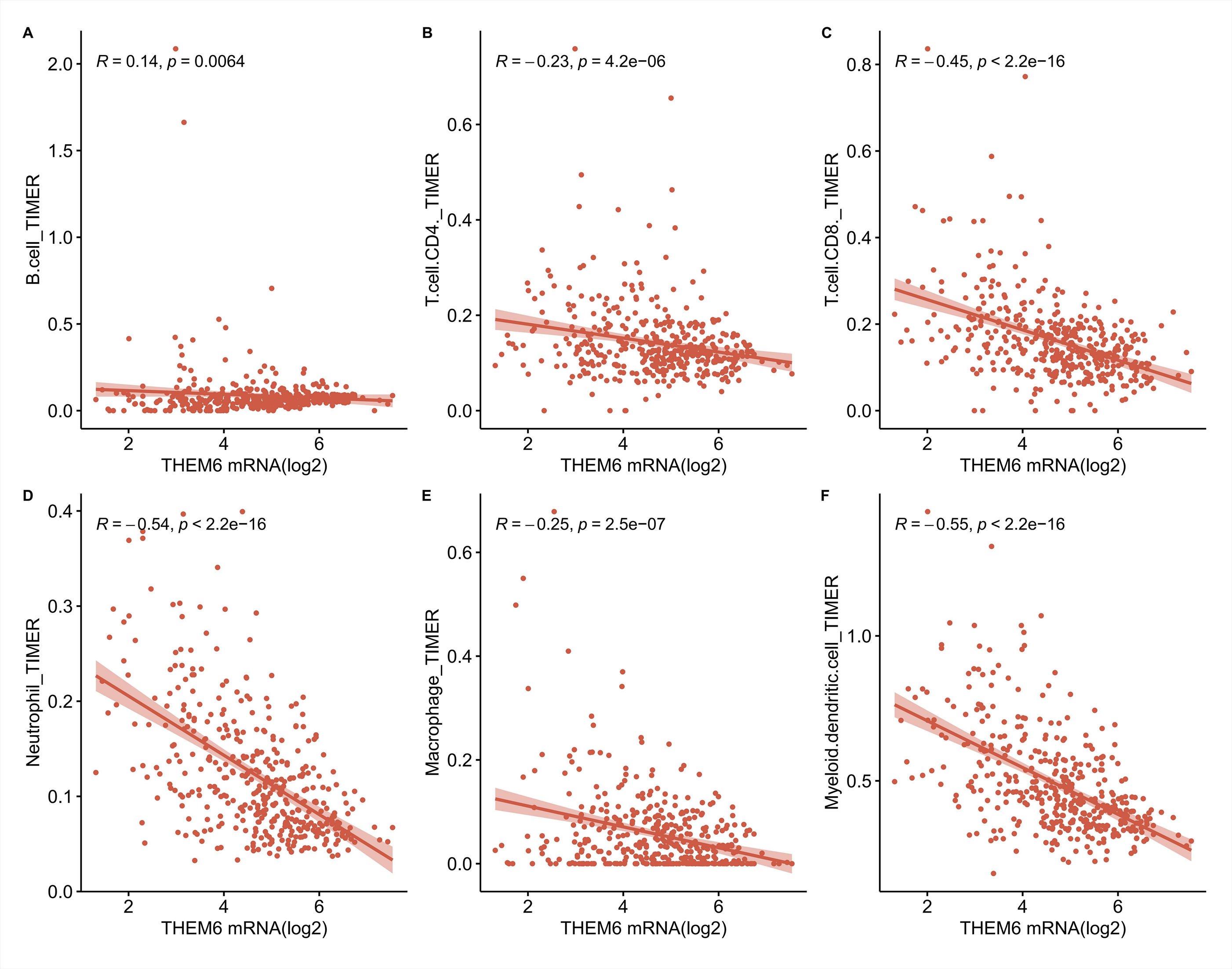

Supplement: Supplementary Materials — Figure S1: the expression pattern and prognostic analysis for overall survival of THEM6 in pan-cancers. (A, B) The expression pattern of THEM6 of pan-cancers in TCGA and GTEx. The asterisks indicated a significant statistical P value calculated with the Mann–Whitney U test (∗P < 0.05; ∗∗P < 0.01; ∗∗∗P < 0.001). (C) The prognostic analyses of THEM6 in pan-cancers using a univariate Cox regression model. Hazard ratio > 1 indicated a risk factor and hazard ratio < 1 represented a protective factor. (D) The prognostic analyses of THEM6 in pan-cancers using the Kaplan-Meier method and log-rank test. Only cancers in which THEM6 was a significant prognostic biomarker were shown. Figure S2: the correlation of THEM6 mRNA expression and immune score in pan-cancers. The P value was calculated by estimating the R page. Figure S3: the correlation of THEM6 mRNA expression and stromal score in pan-cancers. The P value was calculated by estimating the R page. Figure S4: immunological correlation of THEM6 in pan-cancers. Three cancers with the most significant differences in inflammatory cell infiltration in the pan-cancer were identified (including BLCA, BRCA, and LGG). Using the TIMER algorithm, P value calculated with the Mann–Whitney U test. Figures S5–S9: correlations between THEM6 and the tumor-associated immune cells calculated with the QuanTIseq algorithm, CIBERSORT-ABS algorithm, TISIDB algorithm, TIMER algorithm, and TIP algorithm, respectively. The P value was calculated with the Spearman correlation analysis. Figure S10: correlation between THEM6 and 122 immunomodulators in Xiangya cohort. The color and the values indicate the Spearman correlation coefficient. Figures S11–S17: correlations between THEM6 and the tumor-associated immune cells calculated with the TIME algorithm, CIBERSORT-ABS algorithm, QuanTIseq algorithm, xCell algorithm, MCP-counter algorithm, TIP algorithm, and EPIC algorithm, respectively, in the Xiangya cohort. The P value was calculated with the Spea [file 7147279.f1.zip › Figure S8.png]

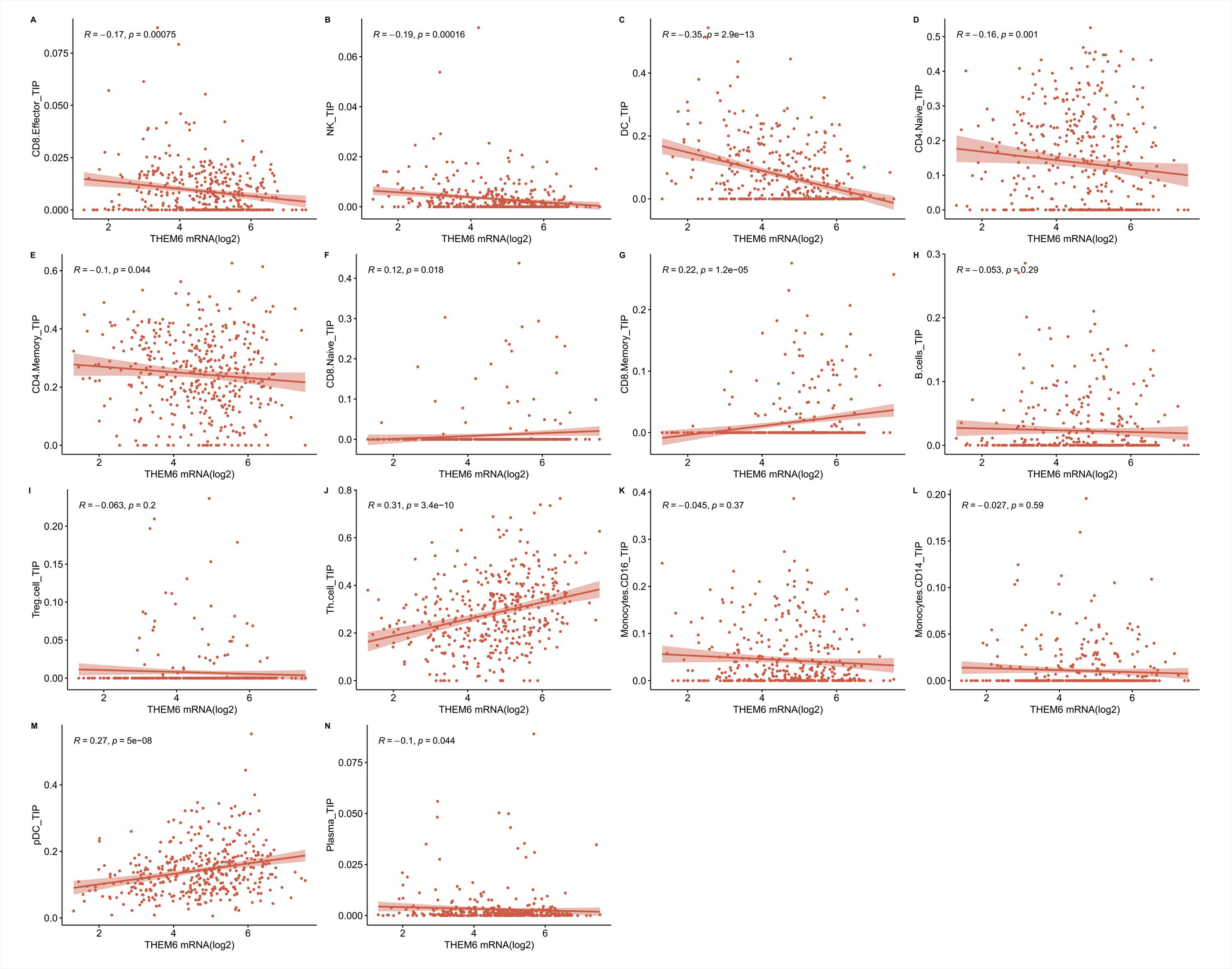

Supplement: Supplementary Materials — Figure S1: the expression pattern and prognostic analysis for overall survival of THEM6 in pan-cancers. (A, B) The expression pattern of THEM6 of pan-cancers in TCGA and GTEx. The asterisks indicated a significant statistical P value calculated with the Mann–Whitney U test (∗P < 0.05; ∗∗P < 0.01; ∗∗∗P < 0.001). (C) The prognostic analyses of THEM6 in pan-cancers using a univariate Cox regression model. Hazard ratio > 1 indicated a risk factor and hazard ratio < 1 represented a protective factor. (D) The prognostic analyses of THEM6 in pan-cancers using the Kaplan-Meier method and log-rank test. Only cancers in which THEM6 was a significant prognostic biomarker were shown. Figure S2: the correlation of THEM6 mRNA expression and immune score in pan-cancers. The P value was calculated by estimating the R page. Figure S3: the correlation of THEM6 mRNA expression and stromal score in pan-cancers. The P value was calculated by estimating the R page. Figure S4: immunological correlation of THEM6 in pan-cancers. Three cancers with the most significant differences in inflammatory cell infiltration in the pan-cancer were identified (including BLCA, BRCA, and LGG). Using the TIMER algorithm, P value calculated with the Mann–Whitney U test. Figures S5–S9: correlations between THEM6 and the tumor-associated immune cells calculated with the QuanTIseq algorithm, CIBERSORT-ABS algorithm, TISIDB algorithm, TIMER algorithm, and TIP algorithm, respectively. The P value was calculated with the Spearman correlation analysis. Figure S10: correlation between THEM6 and 122 immunomodulators in Xiangya cohort. The color and the values indicate the Spearman correlation coefficient. Figures S11–S17: correlations between THEM6 and the tumor-associated immune cells calculated with the TIME algorithm, CIBERSORT-ABS algorithm, QuanTIseq algorithm, xCell algorithm, MCP-counter algorithm, TIP algorithm, and EPIC algorithm, respectively, in the Xiangya cohort. The P value was calculated with the Spea [file 7147279.f1.zip › Figure S9.png]
