# Supplementary material for: THEM6: A Novel Molecular Biomarker Predicts Tumor Microenvironment, Molecular Subtype, and Prognosis in Bladder Cancer
Source: Dis Markers. 2022 Jul 21;2022:7147279. doi: 10.1155/2022/7147279 (PMC9334031; doi:10.1155/2022/7147279)
Supplement: Supplementary Materials — Figure S1: the expression pattern and prognostic analysis for overall survival of THEM6 in pan-cancers. (A, B) The expression pattern of THEM6 of pan-cancers in TCGA and GTEx. The asterisks indicated a significant statistical P value calculated with the Mann–Whitney U test (∗P < 0.05; ∗∗P < 0.01; ∗∗∗P < 0.001). (C) The prognostic analyses of THEM6 in pan-cancers using a univariate Cox regression model. Hazard ratio > 1 indicated a risk factor and hazard ratio < 1 represented a protective factor. (D) The prognostic analyses of THEM6 in pan-cancers using the Kaplan-Meier method and log-rank test. Only cancers in which THEM6 was a significant prognostic biomarker were shown. Figure S2: the correlation of THEM6 mRNA expression and immune score in pan-cancers. The P value was calculated by estimating the R page. Figure S3: the correlation of THEM6 mRNA expression and stromal score in pan-cancers. The P value was calculated by estimating the R page. Figure S4: immunological correlation of THEM6 in pan-cancers. Three cancers with the most significant differences in inflammatory cell infiltration in the pan-cancer were identified (including BLCA, BRCA, and LGG). Using the TIMER algorithm, P value calculated with the Mann–Whitney U test. Figures S5–S9: correlations between THEM6 and the tumor-associated immune cells calculated with the QuanTIseq algorithm, CIBERSORT-ABS algorithm, TISIDB algorithm, TIMER algorithm, and TIP algorithm, respectively. The P value was calculated with the Spearman correlation analysis. Figure S10: correlation between THEM6 and 122 immunomodulators in Xiangya cohort. The color and the values indicate the Spearman correlation coefficient. Figures S11–S17: correlations between THEM6 and the tumor-associated immune cells calculated with the TIME algorithm, CIBERSORT-ABS algorithm, QuanTIseq algorithm, xCell algorithm, MCP-counter algorithm, TIP algorithm, and EPIC algorithm, respectively, in the Xiangya cohort. The P value was calculated with the Spea [file 7147279.f1.zip › Table S2.doc]

Clinicopathological information of Xiangya cohort

| ID | Gender | Age | Grade | AJCCstage |
| --- | --- | --- | --- | --- |
| A01 | Male | 64 | High Grade | Low Stage |
| A03 | Male | 76 | High Grade | High Stage |
| A05 | Male | 67 | High Grade | Low Stage |
| A07 | Male | 82 | High Grade | High Stage |
| A09 | Male | 82 | High Grade | Low Stage |
| A11 | Male | 62 | High Grade | Low Stage |
| B01 | Male | 75 | High Grade | High Stage |
| B03 | Male | 80 | High Grade | Low Stage |
| B05 | Male | 50 | High Grade | High Stage |
| B07 | Male | 59 | High Grade | High Stage |
| B09 | Male | 66 | High Grade | Low Stage |
| B11 | Male | 79 | High Grade | High Stage |
| C01 | Male | 76 | High Grade | High Stage |
| C03 | Male | 67 | High Grade | Low Stage |
| C05 | Male | 83 | High Grade | NA |
| C07 | Male | 81 | Low Grade | Low Stage |
| C09 | Male | 75 | High Grade | High Stage |
| C10 | Male | 71 | High Grade | High Stage |
| C11 | Male | 75 | High Grade | High Stage |
| C12 | Female | 72 | High Grade | Low Stage |
| D01 | Female | 66 | High Grade | Low Stage |
| D02 | Male | 67 | High Grade | High Stage |
| D03 | Male | 58 | High Grade | NA |
| D04 | Male | 77 | High Grade | NA |
| D05 | Male | 68 | High Grade | High Stage |
| D06 | Male | 61 | High Grade | Low Stage |
| D07 | Female | 58 | High Grade | High Stage |
| D08 | Male | 73 | High Grade | NA |
| D09 | Female | 42 | High Grade | High Stage |
| D10 | Male | 57 | High Grade | Low Stage |
| D11 | Male | 55 | High Grade | High Stage |
| D12 | Male | 75 | Low Grade | Low Stage |
| E01 | Male | 73 | High Grade | High Stage |
| E02 | Male | 77 | High Grade | High Stage |
| E03 | Male | 57 | High Grade | Low Stage |
| E04 | Male | 78 | High Grade | NA |
| E05 | Male | 74 | High Grade | Low Stage |
| E06 | Female | 72 | Low Grade | NA |
| E07 | Female | 65 | High Grade | High Stage |
| E08 | Male | 59 | NA | High Stage |
| E09 | Male | 75 | High Grade | High Stage |
| E10 | Male | 55 | High Grade | High Stage |
| E11 | Male | 57 | High Grade | High Stage |
| E12 | Male | 61 | High Grade | High Stage |
| F01 | Male | 79 | High Grade | High Stage |
| F02 | Male | 77 | High Grade | Low Stage |
| F03 | Male | 48 | High Grade | Low Stage |
| F04 | Female |  | NA | NA |
| F05 | Female | 85 | High Grade | High Stage |
| F06 | Male | 76 | High Grade | Low Stage |
| F07 | Male | 61 | Low Grade | Low Stage |
| F08 | Male | 75 | High Grade | Low Stage |
| F09 | Female | 66 | Low Grade | NA |
| F10 | Male | 84 | High Grade | High Stage |
| F11 | Male | 75 | High Grade | High Stage |
| F12 | Male | 78 | Low Grade | Low Stage |
| G01 | Male | 44 | High Grade | High Stage |
| G02 | Female | 77 | High Grade | High Stage |
