# Supplementary material for: THEM6: A Novel Molecular Biomarker Predicts Tumor Microenvironment, Molecular Subtype, and Prognosis in Bladder Cancer
Source: Dis Markers. 2022 Jul 21;2022:7147279. doi: 10.1155/2022/7147279 (PMC9334031; doi:10.1155/2022/7147279)
Supplement: Supplementary Materials — Figure S1: the expression pattern and prognostic analysis for overall survival of THEM6 in pan-cancers. (A, B) The expression pattern of THEM6 of pan-cancers in TCGA and GTEx. The asterisks indicated a significant statistical P value calculated with the Mann–Whitney U test (∗P < 0.05; ∗∗P < 0.01; ∗∗∗P < 0.001). (C) The prognostic analyses of THEM6 in pan-cancers using a univariate Cox regression model. Hazard ratio > 1 indicated a risk factor and hazard ratio < 1 represented a protective factor. (D) The prognostic analyses of THEM6 in pan-cancers using the Kaplan-Meier method and log-rank test. Only cancers in which THEM6 was a significant prognostic biomarker were shown. Figure S2: the correlation of THEM6 mRNA expression and immune score in pan-cancers. The P value was calculated by estimating the R page. Figure S3: the correlation of THEM6 mRNA expression and stromal score in pan-cancers. The P value was calculated by estimating the R page. Figure S4: immunological correlation of THEM6 in pan-cancers. Three cancers with the most significant differences in inflammatory cell infiltration in the pan-cancer were identified (including BLCA, BRCA, and LGG). Using the TIMER algorithm, P value calculated with the Mann–Whitney U test. Figures S5–S9: correlations between THEM6 and the tumor-associated immune cells calculated with the QuanTIseq algorithm, CIBERSORT-ABS algorithm, TISIDB algorithm, TIMER algorithm, and TIP algorithm, respectively. The P value was calculated with the Spearman correlation analysis. Figure S10: correlation between THEM6 and 122 immunomodulators in Xiangya cohort. The color and the values indicate the Spearman correlation coefficient. Figures S11–S17: correlations between THEM6 and the tumor-associated immune cells calculated with the TIME algorithm, CIBERSORT-ABS algorithm, QuanTIseq algorithm, xCell algorithm, MCP-counter algorithm, TIP algorithm, and EPIC algorithm, respectively, in the Xiangya cohort. The P value was calculated with the Spea [file 7147279.f1.zip › Table S3.doc]

80 common differentially expressed RNAs.

CLMP

SFRP2

SPINK1

TNFAIP6

COL3A1

BTBD16

CD163

KRT14

HMGCS2

FCGR2A

COL6A2

S100P

UPK1A

CYP4F23P

FOXQ1

HLA-DRB1

AXL

FPR3

IL32

CAPS

COL5A2

VIM

CLCA4

LYZ

COL6A3

SPP1

CLIC4

FYB1

GZMA

GFPT2

CXCL2

KRT13

TIMP2

FCGR3A

MT1L

UPK2

SULF2

COL6A1

SIRPA

FOXA1

CYP4F12

OSMR

SCNN1B

GRHL3

PMEPA1

F3

TUBB6

SERPING1

GPX2

GBP1

GBP5

CTHRC1

ADAM19

EMP3

IFITM1

PSCA

SLC14A1

CXCL9

MT2A

PLAUR

CYBB

FGFR3

THEMIS2

SLITRK6

CHI3L1

CYP4F8

GATA3-AS1

NNMT

SERPINA1

TYMP

COL16A1

SAA1

FN1

AGR2

IFIT3

DHRS2

MMP11

EEF1A2

FABP6

NKG7
