# Supplementary material for: THEM6: A Novel Molecular Biomarker Predicts Tumor Microenvironment, Molecular Subtype, and Prognosis in Bladder Cancer
Source: Dis Markers. 2022 Jul 21;2022:7147279. doi: 10.1155/2022/7147279 (PMC9334031; doi:10.1155/2022/7147279)
Supplement: Supplementary Materials — Figure S1: the expression pattern and prognostic analysis for overall survival of THEM6 in pan-cancers. (A, B) The expression pattern of THEM6 of pan-cancers in TCGA and GTEx. The asterisks indicated a significant statistical P value calculated with the Mann–Whitney U test (∗P < 0.05; ∗∗P < 0.01; ∗∗∗P < 0.001). (C) The prognostic analyses of THEM6 in pan-cancers using a univariate Cox regression model. Hazard ratio > 1 indicated a risk factor and hazard ratio < 1 represented a protective factor. (D) The prognostic analyses of THEM6 in pan-cancers using the Kaplan-Meier method and log-rank test. Only cancers in which THEM6 was a significant prognostic biomarker were shown. Figure S2: the correlation of THEM6 mRNA expression and immune score in pan-cancers. The P value was calculated by estimating the R page. Figure S3: the correlation of THEM6 mRNA expression and stromal score in pan-cancers. The P value was calculated by estimating the R page. Figure S4: immunological correlation of THEM6 in pan-cancers. Three cancers with the most significant differences in inflammatory cell infiltration in the pan-cancer were identified (including BLCA, BRCA, and LGG). Using the TIMER algorithm, P value calculated with the Mann–Whitney U test. Figures S5–S9: correlations between THEM6 and the tumor-associated immune cells calculated with the QuanTIseq algorithm, CIBERSORT-ABS algorithm, TISIDB algorithm, TIMER algorithm, and TIP algorithm, respectively. The P value was calculated with the Spearman correlation analysis. Figure S10: correlation between THEM6 and 122 immunomodulators in Xiangya cohort. The color and the values indicate the Spearman correlation coefficient. Figures S11–S17: correlations between THEM6 and the tumor-associated immune cells calculated with the TIME algorithm, CIBERSORT-ABS algorithm, QuanTIseq algorithm, xCell algorithm, MCP-counter algorithm, TIP algorithm, and EPIC algorithm, respectively, in the Xiangya cohort. The P value was calculated with the Spea [file 7147279.f1.zip › Table S5.doc]

| Clinical Univariable and Multivariable Cox anlysis in TCGA BLCA cohort |  |  |  |  |  |  |
| --- | --- | --- | --- | --- | --- | --- |
| Variables | Univariate analysis |  |  | Multivariate analysis |  |  |
|  | HR | 95% CI | P | HR | 95% CI | P |
| Age (years) |  |  |  |  |  |  |
| ＜=60 vs >60 | 0.53 | 0.36-0.78 | <0.001 | 0.35 | 0.20-0.62 | <0.001 |
| Gender |  |  |  |  |  |  |
| Male vs Female | 0.86 | 0.62-1.19 | 0.35 | 0.8 | 0.54-1.19 | 0.27 |
| Grade |  |  |  |  |  |  |
| Low vs High | 0.34 | 0.08-1.37 | 0.13 | NA | NA | 0.99 |
| Stage |  |  |  |  |  |  |
| Low vs High | 0.44 | 0.31-0.64 | <0.001 | 0.55 | 0.33-0.93 | 0.03 |
| Subtype |  |  |  |  |  |  |
| Papillary vs Non-Papillary | 0.69 | 0.49-0.98 | 0.04 | 1.08 | 0.70-1.66 | 0.73 |
| LVI |  |  |  |  |  |  |
| Positive vs Negative | 2.33 | 1.60-3.39 | <0.001 | 2.07 | 1.39-3.07 | <0.001 |
|  |  |  |  |  |  |  |
